# Nationally representative prevalence estimates of gay, bisexual, and other men who have sex with men who have served in the U.S. military

**DOI:** 10.1371/journal.pone.0182222

**Published:** 2017-08-01

**Authors:** Karen W. Hoover, Kevin L. Tao, Philip J. Peters

**Affiliations:** Division of HIV/AIDS Prevention, Centers for Disease Control and Prevention (CDC), Atlanta, Georgia, United States of America; University of New South Wales, AUSTRALIA

## Abstract

**Objectives:**

To estimate the number of men in the U.S. military who are gay, bisexual, or other men who have sex with men (MSM) to inform the development of military and other federal policies.

**Study design:**

We analyzed data from the National Surveys of Family Growth to estimate the number of U.S. men who were gay, bisexual, or MSM, and who had served in the military, compared to those who did not serve. We stratified using hierarchical categories of gay, bisexual, and other MSM to compare proportions in the military and general population.

**Results:**

We found that 4.23% of men self-reported as gay, bisexual, or other MSM among men who served in the military, compared to 4.14% among men who had not served (p = 0.93). When stratified, we found that 0.78% self-reported as gay among men who served in the military, compared to 2.12% among men who had not served (p<0.001).

**Conclusions:**

The proportion of men who identified as a gay was lower in the military than in the general population. This finding might have been influenced by historical military policies related to sexual orientation.

## Introduction

Historically, estimates of the number of military personnel who are gay or bisexual have been used to evaluate the impact of the “Don’t Ask, Don’t Tell” (DADT) policy in United States military, and of same-sex marriage benefits for military personnel by the Congressional Budget Office[1–5]. DADT was implemented in 1994 to allow gay and bisexual men and women to serve in the military[6]. But DADT had a negative impact on the health of troops and their communities [7–9]. It was ended with the Don't Ask, Don't Tell Repeal Act of 2010 [7, 10]. Yet the need remains for an accurate estimate of the number of gay and bisexual military personnel for the U.S. Congress and the Department of Defense as they develop policies that effect the rights of and services for this population.

Accurate estimates of the number of military personnel who are gay or bisexual are necessary to deliver optimal health services, including the provision of recommended HIV and STD prevention and care services, as well as other sexual, reproductive, and behavioral health services [8]. In both the U.S. general and military populations, the largest number of new HIV diagnoses and syphilis cases have been in men who have sex with men (MSM) [11–14]. While all military personnel might be at risk of mental health or substance abuse problems, especially those who serve on combat tours, gay and bisexual military personnel have been found to be at increased risk[15–20]. Gay and bisexual military personnel might also experience stigma and victimization while serving[3, 21].

Few studies have estimated the percentage of U.S. military personnel who were gay, bisexual, or other MSM, and methods are needed that provide a direct, simple, and reliable estimate. In this study, we used a nationally representative survey to calculate a direct estimate of the percentage of men who self-reported as gay, bisexual, or other MSM among men aged 18–44 years who had served in the military.

## Materials and methods

We analyzed public use data from the 2002, 2006–2010, and 2011–2013 National Survey of Family Growth (NSFG) that were conducted by National Center for Health Statistics (NCHS) at the Centers for Disease Control and Prevention (CDC). NSFG was designed to include nationally representative multistage area probability samples of the household population of men and women aged 15–44 years in 50 states and the District of Columbia [22, 23]. The informed consent procedures and fieldwork protocol for participant recruitment and interview procedures were reviewed and approved by the Ethics Review Board (ERB) at NCHS and also by the Institutional Review Board of the University of Michigan. Recruitment consisted of 5 steps: (1) a letter and NSFG Question-and-Answer Brochure were mailed to all selected housing units prior to initiating in-person contact; (2) a field interviewer from the University of Michigan visited selected households; (3) a household adult aged 18 years or older was screened using a brief screener to determine if anyone aged 15-44years lived in the household; (4) one person aged 15–44 years was randomly selected from each household; (5) selected persons who agreed to participate were asked to provide an electronic signature acknowledging informed consent, and for selected person was aged 15–17 years both a parent and the minor were required to sign the Parent Permission Form and Minor’s Permission For Interview Form, respectively.

Prior to release of the NSFG public use data by NCHS, the data were reviewed by the NCHS Disclosure Review Board and the NCHS Confidentiality Officer. To minimize the risk of identifying respondents, the data included no personal identifying information such as name and address, and geographic information was restricted to three categories of Metropolitan Statistical Area. The public use data also included several re-coded and re-categorized variables so that respondents could not be identified. None of the authors of this study had access to any personal identifying information.

To generate robust and reliable national estimates, we combined interview data from multiple years. In 2002, 4,928 men were interviewed; in 2006–2010, 10,403 were interviewed; and in 2011–2013, 4,815 were interviewed. The response rates for men were 78%, 75%, and 72%, respectively. Computer-assisted personal interviewing was used to ask most questions, and Audio Computer-Assisted Self-Interviewing (ACASI) was used to ask sensitive questions, including those about sexual orientation and sexual behavior. Only men aged 18–44 years were asked about their previous military service, so we restricted our analysis this gender and age group. We identified those who served in the military with the question, “Have you ever been on active duty in the Armed Forces for a period of 6 months or more?” with response categories of “Yes” or “No”. Respondents were asked about their sexual orientation with the question, “Do you think of yourself as heterosexual, homosexual, bisexual, or something else?” with response categories of “Heterosexual”, “Homosexual”, “Bisexual”, “Something else”, or “Don’t know”. Respondents were hierarchically categorized as gay if they responded “Homosexual” and as bisexual if they responded “Bisexual” to this question. Respondents were also asked about their sexual behavior with the question, “Thinking about the last 12 months, how many male sex partners have you had in the 12 months? Please count every partner, even those you had sex with only once in those 12 months.” If respondents who had not reported their sexual orientation as gay or bisexual but reported ≥ 1 male sex partner(s) were hierarchically categorized as other MSM.

We calculated the proportion of all U.S. men aged 18–44 years who self-reported as gay, bisexual, or other MSM and estimated their demographic and personal characteristics, including age, race and ethnicity, education level, metropolitan statistical area (MSA) of residence, and military service. We also stratified the proportion of U.S. males who were gay, bisexual, or other MSM by NSFG survey cycle.

We also performed bivariate analyses to compare the proportions of men who self-reported as gay, bisexual, or other MSM, respectively, among men who had served in military compared to men who had not served. All analyses were conducted with SAS version 9.2 (SAS Institute, INC., Cary, NC) and SUDAAN version 10.0.1 (Research Triangle Institute, Research Triangle Park, NC) to generate weighted estimates and 95% conference intervals (CI) that account for the complex sampling design of NSFG. Chi-square tests were used to compare differences in proportions, and differences with a two-tailed probability of < 0.05 were considered statistically significant.

## Results

Interviews were conducted with a total of 17,452 men aged 18–44 years in the 2002, 2006–2010, and 2011–2013 NSFG surveys combined. On average, among a weighted 55.2 million men aged 18–44 years, 2,289,234 self-reported as gay, bisexual, or other MSM (4.15%), and when using a hierarchy of gay, bisexual, and other MSM categories 1,102,706 self-reported as gay (2.00%), 910,450 as bisexual (1.65%), and 276,078 as other MSM (0.50%). When stratified by NSFG survey cycle, we found that the percentages of men who reported as gay, bisexual, or other MSM varied: among a weighted 55.4 million men, 2,653,500 (4.79%) self-reported as gay, bisexual, or other MSM in 2002; among 55.5 million men, 1,794,380 (3.23%) in 2006–2010; and among 54.7 million men, 2,419,830 (4.42%) in 2011–2013 (Table 1). The percentages of men who reported as gay, bisexual, or other MSM was significantly lower in 2006–2010 survey cycle than in the 2002 (p = 0.0068) or 2011–2013 survey cycles (p = 0.026). Demographic characteristics of U.S. men who were gay, bisexual, or other MSM are presented in Table 1. About half the men were aged 18–30 years (49.7%); most were white non-Hispanic (62.6%); most attended college or graduate school (59.8%); and most resided in an MSA (86.3%). In response to questions about sexual orientation and behavior, 48.2% self-reported as gay, 39.8% as bisexual, and 12.0% as other MSM.

**Table 1:** Characteristics of men aged 18–44 years who self-reported as gay, bisexual, or other men who have sex with men (MSM) in the United States, National Survey of Family Growth 2002, 2006–2010, and 2011–2013.

Characteristic	N*	%* (95% confidence intervals)
Total	2,289,234	
		
Age group (years)		
18–30	1,138,464	49.7 (44.4–55.1)
31–44	1,150,770	50.3 (44.9–55.6)
Race and ethnicity		
White non-Hispanic	1,433,879	62.6 (57.5–67.5)
Black non-Hispanic	449,407	19.6 (15.7–24.3)
Hispanic	238,882	10.4 (8.2–13.3)
Other	167,066	7.3 (5.1–10.3)
Education (years)		
< 9	158,833	6.9 (5.2–9.3)
9–12	761,303	33.3 (28.3–38.6)
College/graduate school, 1–4	1,030,563	45.0 (39.9–50.3)
College/graduate school, ≥ 5	338,535	14.8 (11.2–19.3)
Metropolitan statistical area (MSA)		
Principal city of MSA	929,235	40.6 (34.3–47.2)
Other MSA	1,046,047	45.7 (39.7–51.8)
Not MSA	303,952	13.7 (9.7–19.1)
Sexual orientation		
Gay	1,102,706	48.2 (42.8–53.6)
BisexualOther MSM	910,450276,078	39.8 (34.4–45.4)12.0 (8.9–16.1)
Military service		
Yes	218,542	9.6 (6.2–14.4)
No	2,070,692	90.4 (85.6–93.8)

*Data were weighted to generate nationally representative estimates

**Fig 1:**
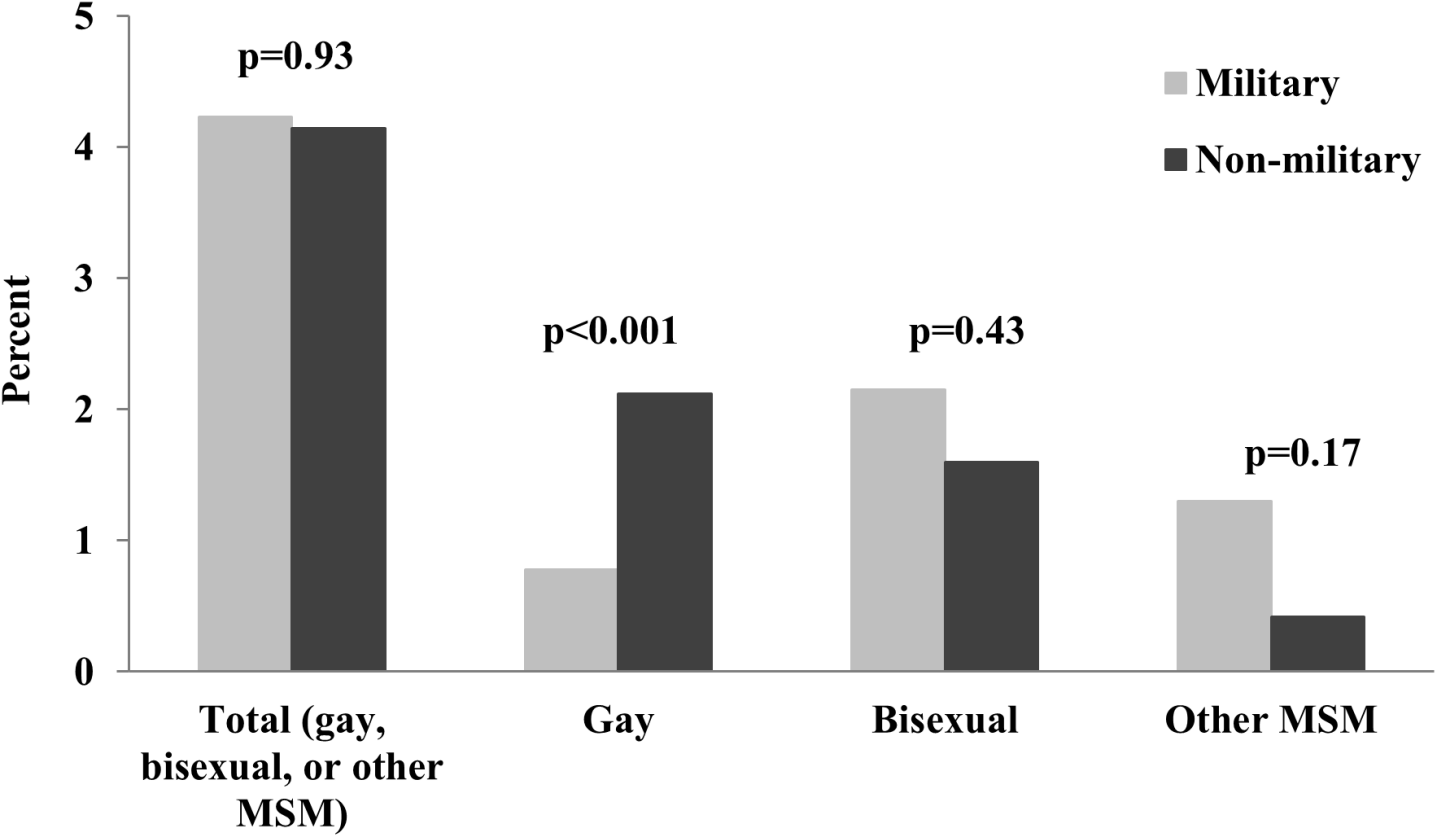
Percentage of men aged 18–44 years who self-reported as gay, bisexual, or other men who have sex with men (MSM) among men who served in the military compared to those who had not served in the military in the United States, National Survey of Family Growth 2002, 2006–2010, 2011–2013.

We found that 4.23% (95% CI: 2.68%–6.62%) of men self-reported as gay, bisexual, or other MSM among men who served in military, compared to 4.14% (95% CI: 3.67%–4.67%) among men who had not served (p = 0.93). Among men who served in military, the percentages who self-reported as gay, bisexual, or other MSM did not differ between the 2002–2010 and 2011–2013 NSFG cycles, with 3.57% (95% CI:2.12%-5.94%) in 2002–2010 and 5.52% (95% CI: 2.47%-11.89%) in 2011–2013 (p = 0.41). However, when stratified using a hierarchy of gay, bisexual, and other MSM categories, we found that 0.78% (95% CI: 0.50%–1.21%) of men self-reported as gay among men who served in the military, compared to 2.12% (95%CI: 1.79%–2.52%) among men who had not served (p<0.001); 2.15% (95% CI: 1.12%–4.09%) as bisexual among men who had served, compared to 1.60% (95% CI: 1.35%–1.89%) among men who had not served (p = 0.43); and 1.30% (95% CI: 0.50%–3.34%) as other MSM among men who had served, compared to 0.42% (95%CI: 0.31%-0.56%) among men who had not served (p = 0.17). (Fig 1).

## Discussion

In our analysis of national interview data, we found that from 2002–2013 on average 4.23% of U.S. men aged 18–44 years who served in the military self-reported as gay, bisexual, or other MSM. Self-reported sexual orientation and sexual behavior using ACASI, with assurance of confidentiality, might provide more reliable measurement of gay, bisexual, or other MSM persons in a population than other methods [24]. Our estimate is higher than previously reported estimates of 1.9% in 2004 and 1.5% in 2010,[2, 5] but likely simple, direct, and reliable because in NSFG men self-reported sexual orientation and sexual behavior using ACASI and sampling was designed to provide nationally representative estimates. Previous estimates were calculated using complicated methods that included U.S. Census data and several national surveys, applied the complex statistical procedure Bayes’ rule, and was based on several assumptions including that 25% of gay men were coupled.

We found a similar percentage of gay, bisexual, or other MSM in the military and U.S. populations, but the percentage of gay men who served in the military was significantly lower than in the general U.S. population. It is possible that men who self-identified as gay were discouraged from serving in the military because they feared discrimination or stigma stemming from the DADT policy. MSM who served in the military might have been less likely to self-report as gay even after they left service, possibly because of concern about losing Veterans Administration (VA) benefits despite confidentiality of NSFG and protected VA benefits for gay and bisexual men and women [25]. With the repeal of the DADT policy in 2011, more gay men might serve and continued monitoring of the percentage of gay and bisexual men in the military will be necessary to understand these trends. Confidential, quality sexual health services are important for all sexually active MSM regardless of their sexual orientation, and our study has provided an updated and accurate estimate of the size of this population. Similar to our findings, a recent analysis of Behavioral Risk Factor Surveillance System data from 10 U.S. states found that military service was reported less frequently by gay and bisexual men than heterosexual men [26].

The military healthcare system provides services to protect the health of its personnel, including HIV prevention and treatment services. Treatment and care of HIV positive personnel is expensive for the military and results in restricted duty assignments of troops,[4] further underscoring the importance of HIV prevention. While the rate of new HIV diagnoses has been low among active duty personnel in the U.S. military,[27] they might be at risk of acquiring HIV and other STDs if they are part of a sexual networks with a high prevalence of these infections [8]. Both HIV positive and HIV negative MSM in the military have been found to engage in ongoing sexual risk behaviors and had high rates of STDs, an objective marker of risk behavior [12, 13, 18]. HIV prevention services, such as preexposure prophylaxis (PrEP), STD testing and treatment, and risk reduction counseling are effective HIV prevention interventions but can only be effectively employed in settings where healthcare providers are aware of their patient’s risk for HIV infection [28, 29] [30]. PrEP with daily oral Truvada is a highly effective HIV prevention method recommended for MSM who are at substantial risk of acquiring HIV infection [31]. A survey conducted in early 2012 found that MSM in the military knew that their sexuality could not be used to negatively impact their careers, yet only 70% were comfortable disclosing their sexual orientation to a military healthcare provider and 57% preferred nonmilitary healthcare providers [32]. The overturning of the DADT policy has created opportunities for targeted HIV prevention messages and better access to HIV prevention services.

Our study had some limitations. First, NSFG questions included in our analysis were asked of only men aged 18–44 years, and NSFG did not include men older than 44 years. NSFG asked men about their sexual orientation and behavior after discharge from the military and did not include current active duty military personnel who lived on military bases or were stationed overseas; the most accurate and current estimates of numbers of gay, bisexual, or MSM would be obtained by asking active duty personnel about their sexual orientation and behavior. However, assuming that the proportion of men who were gay, bisexual, or MSM has been constant over the past several years and across age groups, our estimates can be projected to estimate the current numbers. In addition, compared to sexual behavior change over time, self-reported sexual orientation is more stable among gay and bisexual men [33]. Second, the sample size of men who self-reported as gay, bisexual, or MSM was small, providing sufficient statistical power for only limited stratified analyses. Third, NSFG did not query men about the details of their military service, such as branch or rank. Fourth, while approximately 15% of military personnel were female,[34] NSFG did not query women about their military service so we could not estimate the percentage of gay or bisexual women in the military. Fifth, the terms “homosexual” or “bisexual” terms used by NSFG might not have been synonymous with “gay men” or “lesbian, gay, or bisexual (LGB)” used in the other studies. Finally, NSFG did not include questions about depression, mental health, suicidal ideation, or suicide attempt that are prevalent health issues in the military. A recent study showed that LGB veterans were at higher risk of lifetime suicidal ideation than heterosexual veterans [35]. It is important to assess troops and veterans for risk of suicide, especially gay, bisexual, and other MSM, and to implement culturally-sensitive interventions for prevention of suicide.

It might be challenging for a military medical system to provide all the necessary clinical services to meet the unique needs of gay, bisexual, and transgender personnel [29]. But with recent policies that permit people to serve openly, an opportunity exists for the military medical system to provide culturally competent HIV prevention, sexual health, and mental health services required for persons in these populations to achieve optimal health. Simple and reliable estimation of the numbers of gay, bisexual, and transgender military personnel will inform policies and procedures that ensure that the military operates healthcare systems with adequate capacity to meet the clinical needs of this population, and to support efforts to promote equality. Analyses of NSFG data provide a simple, direct, and reliable method to estimate these numbers.

## Supporting information

S1 File nsfg_2011–2013_malesectionj_crq for military services.pdf. (PDF)

S2 File nsfg_2011–2013_male sexual behavior and orientation.pdf. (PDF)
