# Supplementary material for: Nationally representative prevalence estimates of gay, bisexual, and other men who have sex with men who have served in the U.S. military
Source: PLoS One. 2017 Aug 1;12(8):e0182222. doi: 10.1371/journal.pone.0182222 (PMC5538666; doi:10.1371/journal.pone.0182222)
Supplement: S1 File — (PDF) [file pone.0182222.s001.pdf]

SECTION J

Residence and place of birth; Religion; Military service; Past and current work (R and current wife/partner); Attitudes

VARIABLES IMPORTED FROM EARLIER SECTIONS:

**CMINTVW**	**CENTURY MONTH OF THE DATE OF THE INTERVIEW (SECTION A)**
**CMLSTYR_FILL:**	**FILL FOR CENTURY MONTH OF MONTH OF INTERVIEW - 1 YR (FROM A)**
**CMBIRTH**	**CENTURY MONTH OF BIRTHDAY (SECTION A)**
**AGESCRN**	**AGE OF R AT SCREENER (SECTION A)**
**MARSTAT**	**MARITAL STATUS (SECTION A)**
**SEXSTAT**	**DESCRIPTION OF R SEXUAL EXPERIENCE (SECTION B)**
**CWPNAME**	**NAME OF WIFE OR COHABITING PARTNER (SECTION C)**
**ANYKIDS**	**WHETHER R EVER HAD BIO OR ADOPTED KIDS (SECTION F)**

VARIABLES CREATED IN THIS SECTION & OUTPUT TO DATA FILE

**CMSTRUS**	**CENTURY MONTH FOR DATE R CAME TO THE UNITED STATES (FC J-2)**
**CMBEGMIL**	**CENTURY MONTH FOR DATE BEGAN MILITARY SERVICE (FC J-10)**
**CMENDMIL**	**CENTURY MONTH FOR DATE ENDED MILITARY SERVICE (FC J-12)**
**RWRKST**	**R WORK STATUS (BASED ON CURRENT STATUS QUESTION) (FC J-15)**
**WORKP12**	**WHETHER R WORKED IN THE PREVIOUS 12 MONTHS (BASED ON RETROSPECTIVE QUESTIONS) (FC J-16)**
**REARNTY**	**WHETHER R IS CURRENTLY WORKING, OR EVER WORKED AT ALL (FC J-19)**
**SPWRKST**	**WHETHER SPOUSE/PARTNER WAS EMPLOYED (WORKING OR TEMPORARILY NOT WORKING) (FC J-21).**
**SPEARNTY**	**WHETHER SPOUSE/PARTNER IS CURRENTLY WORKING OR EVER WORKED (FC J-24)**
**PMARCOHB**	**WHETHER R EVER COHABITED PREMARITALLY (FC J-26b)**

VARIABLES CREATED IN THIS SECTION & ARE NOT OUTPUT TO DATA FILE:
NONE

Residence and Place of Birth (JA)

{ ASKED FOR ALL
SAMEADD
JA-0. Now I have some questions about where you live.

Were you living at this same address on April 1, 2010?

Yes.................................1
No..................................5

FLOW CHECK J-1: IF JA-0 SAMEADD = 5 (NO), DK/RF, ASK JA-1 CNTRY10.
ELSE IF JA-0 SAMEADD = 1 (YES), GO TO JA-7 BRNOUT.

{ ASKED IF NOT LIVING AT THIS ADDRESS ON APRIL 1, 2010
CNTRY10
JA-1. Were you living in the United States on April 1, 2010?

[HELP AVAILABLE]

Yes.................................1
No..................................5 (BRNOUT JA-7)

ASTATE
JA-4. Please tell me in which state you were living on April 1, 2010.

- *ENTER* state

TYPE: String [20] {VALUE RETURNS FROM STATE DATABASE LINK}

{ ASKED FOR ALL }
BRNOUT
JA-7. Were you born outside of the United States?

[HELP AVAILABLE]

Yes.................................1
No..................................5

FLOW CHECK J-1a: IF JA-7 BRNOUT = 1 (YES), ASK JA-8m STRUS_M.
ELSE, GO TO JB-1 RELRSD.

{ ASKED IF BORN OUTSIDE THE U.S.
STRUS_M
1 of 2
JA-8m. In what month and year did you come to the United States to stay?

- *ENTER MM/YYYY*
- *PROBE for season if DK month.*
- *If R moved out of the U.S. and then moved back, record the date he last moved back here.*

[HELP AVAILABLE]

1. January	5. May	9. September	13. Winter
2. February	6. June	10. October	14. Spring
3. March	7. July	11. November	15. Summer
4. April	8. August	12. December	16. Fall

STRUS_Y
2 of 2
JA-8y. (In what month and year did you come to the United States to stay)?

- *ENTER* (STRUS_M)*/YYYY*

[HELP AVAILABLE]

UNDERLYING RANGE: 1966 to 2020

FLOW CHECK J-2: COMPUTE CMSTRUS (CENTURY MONTH FOR DATE R CAME TO THE US).

SET CMSTRUS = NULL/BLANK.
IF STRUS_Y = RF, THEN CMSTRUS = 9998.
ELSE IF STRUS_Y = DK, THEN CMSTRUS = 9999.
ELSE IF STRUS_M = DK/RF, THEN CMSTRUS = (((STRUS_Y - 1900) * 12) + 6).
ELSE IF STRUS_M = 13, 14, 15 OR 16 USE MONTH APPROPRIATE TO SEASON (MONTH), THEN CMSTRUS = (((STRUS_Y - 1900) * 12) + MONTH).
ELSE CMSTRUS = (((STRUS_Y - 1900) * 12) + STRUS_M).

Edit Check JA8_1: IF (STRUS_M LE 12) AND CMSTRUS > CMINTVW, DISPLAY:
The date entered cannot be after the date of interview. Please correct.
HARD, NONSUPPRESSIBLE EDIT CHECK.

Edit Check JA8_2: **IF (STRUS_M LE 12) AND CMSTRUS < CMBIRTH, DISPLAY:** *The date entered cannot be before his date of birth. Please correct.*
HARD, NONSUPPRESSIBLE EDIT CHECK.

Edit Check JA8_3: **IF (13 LE STRUS_M LE 16) AND CMSTRUS > (CMINTVW + 2), DISPLAY:** *The year entered cannot be after the date of interview. Please correct.*
HARD, NONSUPPRESSIBLE EDIT CHECK.

Edit Check JA8_4: **IF (13 LE STRUS_M LE 16) AND CMSTRUS < (CMBIRTH - 3), DISPLAY:** *The date entered cannot be before his date of birth. Please correct.*
HARD, NONSUPPRESSIBLE EDIT CHECK.

Edit Check JA8_5: **IF (STRUS_M = DK/RF) AND STRUS_Y > ((CMINTVW / 12) + 1900), DISPLAY:** *The date entered cannot be after the date of interview. Please correct.*
HARD, NONSUPPRESSIBLE EDIT CHECK.

Edit Check JA8_6: **IF (STRUS_M = DK/RF) AND STRUS_Y < (((CMBIRTH / 12) + 1900) – 1), DISPLAY:** *The date entered cannot be before his date of birth. Please correct.*
HARD, NONSUPPRESSIBLE EDIT CHECK.

Religion (JB)

{ ASKED FOR ALL
RELRSD
JB-1. Now I have a few questions about religion. Please look at Card 77. In what religion were you raised, if any?

◆*If R says Protestant, ask:* What is the complete name of the denomination? *If necessary, enter [11]*

◆*If R indicates that he was raised in more than one religion, enter the number of the first one mentioned and insert an [F2] comment with the code for the 2nd religion with R's comments.*

◆*ENTER [1] if R was raised "atheist" or "agnostic".*

[SHOW CARD 77]
[HELP AVAILABLE]

None	1
Catholic	2
Jewish	3
Southern Baptist	4
Baptist	5
Methodist or African Methodist	6
Lutheran	7
Presbyterian	8
Episcopal or Anglican	9
Church of Jesus Christ of Latter Day Saints (LDS/Mormon)	10
Other	11

FLOW CHECK J-3: **IF JB-1 RELRSD = 11, ASK JB-2 RELRSD1.**
ELSE GO TO FLOW CHECK J-5.

RELRSD1
JB-2. Please look at Card 78. In what religion were you raised?

[SHOW CARD 78]
[HELP AVAILABLE]

Assemblies of God....................................12
Church of Nazarene...................................13
The Church of God....................................14
The Church of God (Cleveland, TN)....................15
The Church of God in Christ..........................16
7th Day Adventist 17
United Pentecostal Church............................18
Pentecostal Assemblies...............................19
Jehovah's Witness....................................20
Christian, another denomination not listed...........21
Christian, no specific denomination..................22
Unitarian-Universalist...............................23
Greek Orthodox.......................................24
Other Orthodox 25
Muslim...26
Buddhist...27
Hindu..28
Other (specify)......................................29

FLOW CHECK J-4: IF JB-2 RELRSD1 = 29, ASK JB-3 OTHRLRSD.
ELSE GO TO FLOW CHECK J-5.

OTHRLRSD
JB-3. Please tell me the name of the religion in which you were raised.

TYPE: STRING [25]

FLOW CHECK J-5: IF AGESCRN LT 25, ASK JB-4 ATTND14.
ELSE IF AGESCRN GE 25, GO TO JB-5 RELNOW.

{ ASKED IF R IS UNDER AGE 25
ATTND14
JB-4. Please look at Card 79. When you were 14, about how often did you usually attend religious services?

[SHOW CARD 79]
[HELP AVAILABLE]

More than once a week................................1
Once a week..2
2-3 times a month....................................3
Once a month (about 12 times a year).................4
3-11 times a year....................................5
Once or twice a year.................................6
Never..7

{ ASKED FOR ALL
RELNOW
JB-5. Please look at Card 77. What religion are you now, if any?

◆*If R says Protestant, ask:* What is the complete name of

the denomination? *If necessary, enter [11].*

- *If R identifies with more than one religion enter the number of the first one mentioned and insert an [F2] comment with the code for the 2nd religion with R's comments.*

- *ENTER [1] if R is "atheist" or "agnostic".*

[SHOW CARD 77]

None...1
Catholic...2
Jewish...3
Southern Baptist...4
Baptist..5
Methodist or African Methodist...........................6
Lutheran...7
Presbyterian...8
Episcopal or Anglican....................................9
Church of Jesus Christ of Latter Day Saints (LDS/Mormon)..10
Other 11

FLOW CHECK J-6: IF JB-5 RELNOW = 11 THEN ASK JB-6 RELNOW1.
ELSE GO TO FLOW CHECK J-8.

RELNOW1
JB-6. Please look at Card 78. What religion are you now?

[SHOW CARD 78]

Assemblies of God................................12
Church of Nazarene...............................13
The Church of God................................14
The Church of God (Cleveland, TN)................15
The Church of God in Christ......................16
7th Day Adventist 17
United Pentecostal Church........................18
Pentecostal Assemblies...........................19
Jehovah's Witness................................20
Christian, another denomination not listed.......21
Christian, no specific denomination..............22
Unitarian-Universalist...........................23
Greek Orthodox...................................24
Other Orthodox 25
Muslim...26
Buddhist...27
Hindu..28
Other (specify)..................................29

FLOW CHECK J-7: IF JB-6 RELNOW1 = 29, ASK JB-7 OTHRLNOW.
ELSE GO TO FLOW CHECK J-8.

OTHRLNOW
JB-7. Please tell me the name of the religion you are now.

TYPE: STRING [25]

FLOW CHECK J-8: IF JB-6 RELNOW = 1 (None), GO TO JB-10 ATTNDNOW.

ELSE IF JB-6 RELNOW = DK/RF, GO TO JB-9 RELDLIFE.
ELSE IF JB-6 RELNOW = 3 (Jewish) OR RELNOW1 = 26 (Muslim) OR RELNOW1 = 27 (Buddhist) OR RELNOW1 = 28 (Hindu), GO TO JB-9 RELDLIFE
ELSE ASK JB-8 FUNDAM.

FUNDAM
JB-8. Please look at Card 80. Which of these do you consider yourself to be, if any?

- *ENTER all that apply [1-4]*

- *Press [Space] or [-] to separate responses*

[SHOW CARD 80]

A born again Christian..............................1
A charismatic.......................................2
An evangelical......................................3
A fundamentalist 4
None of the above...................................5

EDIT CHECK JB8_1: **IF FUNDAM = 5 AND ANY OTHER CODE, DISPLAY TEXT:** "None of the above" may not be used in combination with any other answer for this question. Verify the answer with R and re-enter."
HARD, NONSUPPRESSIBLE EDIT CHECK.

RELDLIFE
JB-9. Currently, how important is religion in your daily life? Would you say it is very important, somewhat important, or not important?

[HELP AVAILABLE]

Very important......................................1
Somewhat important..................................2
Not important.......................................3

{ ASKED FOR ALL
ATTNDNOW
JB-10. Please look at Card 79. About how often do you attend religious services?

- *If R has difficulty answering, have him think in terms of the past year, and try to give an <u>average</u> frequency of attendance.*

[SHOW CARD 79]
[HELP AVAILABLE]

More than once a week..............................1
Once a week..2
2-3 times a month..................................3
Once a month (about 12 times a year)...............4
3-11 times a year..................................5
Once or twice a year...............................6
Never..7

Military Service (JC)

FLOW CHECK J-9: IF AGESCRN GE 18, ASK JC-1 MILSVC.
ELSE GO TO JD-4 WRK12MOS.

{ ASKED IF R WAS 18 OR OLDER WHEN SCREENED
MILSVC
JC-1. Have you ever been on active duty in the Armed Forces for a period of 6 months or more?

Yes..................................1
No...................................5 (JD-4 WRK12MOS)

BEGMIL_M
1 of 2
JC-2m. In what month and year did that period of active duty begin?

◆*ENTER MM/YYYY*

◆*PROBE for season if DK month*

1. January	5. May	9. September	13. Winter
2. February	6. June	10. October	14. Spring
3. March	7. July	11. November	15. Summer
4. April	8. August	12. December	16. Fall

BEGMIL_Y
2 of 2
JC-2y. (In what month and year did that period of active duty begin?)

◆*ENTER* (BEGMIL_M)*/YYYY*

UNDERLYING RANGE: 1983 to 2020

FLOW CHECK J-10: COMPUTE CMBEGMIL (CENTURY MONTH FOR DATE BEGAN MILITARY SERVICE)

SET CMBEGMIL = NULL/BLANK.
IF BEGMIL_Y = RF, THEN CMBEGMIL = 9998.
ELSE IF BEGMIL_Y = DK, THEN CMBEGMIL = 9999.
ELSE IF BEGMIL_M = DK/RF, THEN CMBEGMIL = (((BEGMIL_Y - 1900) * 12) + 6).
ELSE IF BEGMIL_M = 13, 14, 15 OR 16 USE MONTH APPROPRIATE TO SEASON (MONTH), THEN CMBEGMIL = (((BEGMIL_Y - 1900) * 12) + MONTH).
ELSE CMBEGMIL = (((BEGMIL_Y - 1900) * 12) + BEGMIL_M).

Edit Check JC2_1: **IF (BEGMIL_M LE 12) AND CMBEGMIL > CMINTVW, DISPLAY:** *The date entered cannot be after the date of interview. Please correct.*
HARD, NONSUPPRESSIBLE EDIT CHECK.

Edit Check JC2_2: **IF (BEGMIL_M LE 12) AND CMBEGMIL < CMBIRTH, DISPLAY:** *The date entered cannot be before his date of birth. Please correct.*
HARD, NONSUPPRESSIBLE EDIT CHECK.

Edit Check JC2_3: **IF (13 LE BEGMIL_M LE 16) AND CMBEGMIL > (CMINTVW + 2), DISPLAY:** *The date entered cannot be after the*

date of interview. Please correct.
HARD, NONSUPPRESSIBLE EDIT CHECK.

Edit Check JC2_4: **IF (13 LE BEGMIL_M LE 16) AND CMBEGMIL < (CMBIRTH - 3), DISPLAY:** *The date entered cannot be before his date of birth. Please correct.*
HARD, NONSUPPRESSIBLE EDIT CHECK.

Edit Check JC2_5: **IF (BEGMIL_M = DK/RF) AND BEGMIL_Y > ((CMINTVW / 12) + 1900), DISPLAY:** *The date entered cannot be after the date of interview. Please correct.*
HARD, NONSUPPRESSIBLE EDIT CHECK.

Edit Check JC2_6: **IF (BEGMIL_M = DK/RF) AND BEGMIL_Y < (((CMBIRTH / 12) + 1900) – 1), DISPLAY:** *The date entered cannot be before his date of birth. Please correct.*
HARD, NONSUPPRESSIBLE EDIT CHECK.

EDIT CHECK JC2_7: **IF BEGMIL_Y <> NONRESPONSE AND BEGMIL_M >= 1 AND BEGMIL_M <= 12 AND (CMBEGMIL - CMBIRTH) <= 203, DISPLAY:** *The date entered is before his 17th birthday. Please double check this date and correct or continue.*
SOFT EDIT CHECK, CAN BE SUPPRESSED.

Edit Check JC2_8: **IF BEGMIL_Y <> NONRESPONSE AND BEGMIL_M >= 13 AND BEGMIL_M <= 16 AND (CMBEGMIL – CMBIRTH) <= 200, DISPLAY:** *The date entered may be before his 18th birthday. Please double check this date and correct or continue.*
SOFT EDIT CHECK, CAN BE SUPPRESSED.

Edit Check JC2_9: **IF BEGMIL_Y <> NONRESPONSE AND BEGMIL_M = NONRESPONSE AND (CMBEGMIL – CMBIRTH) <= 197, DISPLAY:** *The date entered may be before his 17th birthday. Please double check this date and correct or continue.*
SOFT EDIT CHECK, CAN BE SUPPRESSED.

ENDMIL_M
1 of 2

JC-3m. What was the month and year of your last separation from active duty?

- *ENTER MM/YYYY*
- *PROBE for season if DK month.*
- *If R is still on active duty, enter [96]*

1. January	5. May	9. September	13. Winter
2. February	6. June	10. October	14. Spring
3. March	7. July	11. November	15. Summer
4. April	8. August	12. December	16. Fall

96. *If vol: R still on active duty.*

FLOW CHECK J-11: IF JC-3 ENDMIL_M = 96, GO TO FLOW CHECK J-12.

ENDMIL_Y

2 of 2

JC-3y. (What was the month and year of your last separation from active duty?)

◆*ENTER* (ENDMIL_M)*/YYYY*

UNDERLYING RANGE: 1983 to 2020

FLOW CHECK J-12: COMPUTE CMENDMIL (CENTURY MONTH FOR DATE ENDED MILITARY SERVICE).

SET CMENDMIL = NULL/BLANK.
IF ENDMIL_Y = RF, THEN CMENDMIL = 9998.
ELSE IF ENDMIL_Y = DK, THEN CMENDMIL = 9999.
ELSE IF ENDMIL_M = 96, THEN CMENDMIL = 9996.
ELSE IF ENDMIL_M = DK/RF, THEN CMENDMIL = (((ENDMIL_Y - 1900) * 12) + 6).
ELSE IF ENDMIL_M = 13, 14, 15 OR 16, USE MONTH APPROPRIATE TO SEASON (MONTH), THEN CMENDMIL = (((ENDMIL_Y - 1900) * 12) + MONTH).
ELSE CMENDMIL = (((ENDMIL_Y - 1900) * 12) + ENDMIL_M).

Edit Check JC3_1: IF (ENDMIL_M LE 12) AND CMENDMIL > CMINTVW, DISPLAY: *The date entered cannot be after the date of interview. Please correct.*
HARD, NONSUPPRESSIBLE EDIT CHECK.

Edit Check JC3_2: IF (ENDMIL_M LE 12) AND CMENDMIL < CMBIRTH, DISPLAY: *The date entered cannot be before his date of birth. Please correct.*
HARD, NONSUPPRESSIBLE EDIT CHECK.

Edit Check JC3_3: IF (13 LE ENDMIL_M LE 16) AND CMENDMIL > (CMINTVW + 2), DISPLAY: *The date entered cannot be after the date of interview. Please correct.*
HARD, NONSUPPRESSIBLE EDIT CHECK.

Edit Check JC3_4: IF (13 LE ENDMIL_M LE 16) AND CMENDMIL < (CMBIRTH - 3), DISPLAY: *The date entered cannot be before his date of birth. Please correct.*
HARD, NONSUPPRESSIBLE EDIT CHECK.

Edit Check JC3_5: IF (ENDMIL_M = DK/RF) AND ENDMIL_Y > ((CMINTVW / 12) + 1900), DISPLAY: *The date entered cannot be after the date of interview. Please correct.*
HARD, NONSUPPRESSIBLE EDIT CHECK.

Edit Check JC3_6: IF (ENDMIL_M = DK/RF) AND ENDMIL_Y < (((CMBIRTH / 12) + 1900) – 1), DISPLAY: *The date entered cannot be before his date of birth. Please correct.*
HARD, NONSUPPRESSIBLE EDIT CHECK.

EDIT CHECK JC3_7: IF CMENDMIL LT CMBEGMIL + 6 MONTHS, DISPLAY: *R has reported an end date for active duty that is less than 6 months after his start date. If these dates are correct, return to JC-1 MILSVC and correct.*
HARD, NONSUPPRESSIBLE EDIT CHECK.
INVOLVING (MILSVC, BEGMIL_M, BEGMIL_Y, ENDMIL_M, ENDMIL_Y)

EDIT CHECK JC3_8: **IF CMENDMIL LT CMBEGMIL, DISPLAY:** *R has reported an end date for active duty that is before his start date. Please correct the incorrect date.*
HARD, NONSUPPRESSIBLE EDIT CHECK.
INVOLVING(MILSVC,BEGMIL_M,BEGMIL_Y,ENDMIL_M,ENDMIL_Y)

Work (JD)

{ ASKED FOR ALL
WRK12MOS
JD-4. Now I'd like to ask about your work experience in the last 12 months. By work, I mean any job for pay that was regularly scheduled, that you were expected to perform. Please include full-time, part-time, and temporary or summer jobs.

In the last 12 months, that is since [CMLSTYR_FILL], for how many months did you have any job for pay?

[HELP AVAILABLE]

- *Active duty military is considered full-time employment/work*
- *ENTER number of months*

UNDERLYING RANGE: 0 to 12

FLOW CHECK J-14: IF JD-4 WRK12MOS = 0, DK/RF, GO TO JE-1 DOLASTWK.

{ ASKED IF R WORKED 1-12 MONTHS
FPT12MOS
JD-5. In the last 12 months, did you work all full-time, all part-time or some of each?

- *Active duty military is considered full-time employment/work*

[HELP AVAILABLE]

Full-time............1
Part time............2
Some of each.........3

Current/Last Job Series (JE)

{ ASKED FOR ALL
DOLASTWK
JE-1. Please look at Card 82. Last week, what were you doing? Were you working, keeping house, going to school, or something else?

- *ENTER all that apply.*

- *"Last week" refers to the week ending the Saturday before the interview.*

- *Active duty military is considered full-time employment/work*

- *Press [Space] or [-] to separate responses*

[SHOW CARD 82]
[HELP AVAILABLE]

Working 1
Not working at job due to temporary illness,
vacation, strike, etc 2
On paternity or family leave from job 3
Unemployed, laid off, or looking for work 4
Keeping house 5
Taking care of family 6
Going to school 7
On permanent disability 8
Something else 9

FLOW CHECK J-15: COMPUTE RWRKST (R CURRENT WORK STATUS)

SET RWRKST = NULL/BLANK.
IF JE-1 DOLASTWK = 1, 2 OR 3, THEN RWRKST = 1 (YES).
ELSE IF JE-1 DOLASTWK = 4, 5, 6, 7, 8, 9, DK/RF, THEN RWRKST = 5 (NO).

FLOW CHECK J-16: COMPUTE WORKP12 (WHETHER R WORKED IN THE PREVIOUS 12 MONTHS)

SET WORKP12 = NULL/BLANK
IF JD-4 WRK12MOS > 0 AND JD-4 WRK12MOS <= 12, THEN WORKP12 = 1 (YES).
ELSE WORKP12 = 5 (NO).

FLOW CHECK J-17: IF RWRKST = 5 (NO) AND WORKP12 = 5 (NO), ASK JE-2 RPAYJOB.
ELSE IF RWRKST = 5 (NO) AND WORKP12 = 1 (YES), GO TO JE-3 RNUMJOB.
ELSE IF RWRKST = 1 (YES) THEN GO TO JE-3 RNUMJOB.

{ ASKED IF R DIDN’T WORK IN THE LAST 12 MONTHS
{ AND WASN’T WORKING LAST WEEK

RPAYJOB

JE-2. Did you ever work at a job or business for pay on a regular basis?

Yes....................1
No.....................5 (FLOW CHECK J-19)

FLOW CHECK J-18: **IF RPAYJOB = DK/RF, THEN GO TO FLOW CHECK J-19.**

RNUMJOB

JE-3. IF JE-1 DOLASTWK = 1 (WORKING), ASK: How many jobs did you work last week?

ELSE IF JE-1 DOLASTWK = 2 or 3 or JE-2 RPAYJOB = 1 or (RWRKST = 5 (NO) and WORKP12 = 1 (YES)), ASK:
How many jobs did you work during the last week you worked?

◆*ENTER number of jobs*

UNDERLYING RANGE: 1 to 6

RFTPTX

JE-4. IF JE-1 DOLASTWK = 1 AND JE-3 RNUMJOB = 1, ASK:
Do you work part time or full time, or some of each? By full time I mean 35 or more hours a week.

ELSE IF JE-1 DOLASTWK = 1 AND (JE-3 RNUMJOB > 1 OR JE-3 RNUMJOB = DK/RF), ASK:
At your primary job, do you work part-time or full-time, or some of each? By full-time I mean 35 or more hours a week.

ELSE IF (JE-1 DOLASTWK = 2 OR 3 or JE-2 RPAYJOB = 1 OR (RWRKST = 5 (NO) and WORKP12 = 1 (YES))) AND JE-3 RNUMJOB = 1, ASK:
Please think about the last week you worked on your job. Did you work part-time or full-time, or some of each? By full-time I mean 35 or more hours a week.

ELSE IF (JE-1 DOLASTWK = 2 OR 3 or JE-2 RPAYJOB = 1 OR (RWRKST = 5 (NO) and WORKP12 = 1 (YES))) AND (JE-3 RNUMJOB > 1 OR JE-3 RNUMJOB = DK/RF), ASK:
Please think about the last week you worked on your primary job. Did you work part-time or full-time, or some of each? By full-time I mean 35 or more hours a week.

Full time...............1
Part time...............2
Some of each............3

FLOW CHECK J-19: COMPUTE REARNTY (WHETHER R EVER WORKED)

SET REARNTY = NULL/BLANK
IF RWRKST = 1 (YES) OR WORKP12 = 1 (YES) OR RPAYJOB = 1 (YES), THEN REARNTY = 1 (YES).
ELSE REARNTY = 5 (NO).

Spouse/Partner’s Current/Last Job Series (JF)

{ ASKED IF R IS CURRENTLY MARRIED OR COHABITING AB-1 MARSTAT = 1 or 2
SPLSTWK
JF-1. Please look at card 81. Last week, what was (CWPNAME) doing? Was she working, keeping house, going to school, or something else?

- ENTER all that apply.

- “Last week” refers to the week ending the Saturday before the interview.

- *Active duty military is considered full-time employment/work*

- *Press [Space] or [-] to separate responses*

[SHOW CARD 81]
[HELP AVAILABLE]

Working...................................... 1
Not working at job due to temporary illness, vacation, strike, etc..................... 2
On maternity or family leave from job......... 3
Unemployed, laid off, or looking for work..... 4

Keeping house................................ 5
Taking care of family 6
Going to school.............................. 7
On permanent disability...................... 8
Something else 9

FLOW CHECK J-21: COMPUTE SPWRKST (SPOUSE/PARTNER'S WORK STATUS).

SET SPWRKST = NULL/BLANK.
IF JF-1 SPLSTWK = 1, 2 OR 3 THEN SPWRKST = 1 (YES).
ELSE IF JF-1 SPLSTWK = 4, 5, 6, 7, 8, 9, DK/RF, THEN SPWRKST = 5 (NO).

FLOW CHECK J-22: IF SPWRKST = 5 (NO), ASK JF-2 SPPAYJOB.
ELSE GO TO JF-3 SPNUMJOB.

{ ASKED IF WIFE/PARTNER NOT EMPLOYED/WORKING LAST WEEK
SPPAYJOB
JF-2. Did she ever work at a job or business for pay on a regular basis?

- *Active duty military is considered full-time employment/work*

Yes.....................1
No......................5 (FLOW CHECK J-24)

FLOW CHECK J-23: IF JF-2 SPPAYJOB = DK/RF, GO TO FLOW CHECK J-24.

{ ASKED IF R'S WIFE/PARTNER WAS WORKING LAST WEEK OR SHE EVER WORKED FOR PAY
SPNUMJOB
JF-3. IF JF-1 SPLSTWK = 1, ASK:
How many jobs did she work last week?

ELSE IF (JF-1 SPLSTWK = 2 or 3) OR JF-2 SPPAYJOB = 1, ASK:
How many jobs did she work during the last week she worked?

- *ENTER number of jobs*

UNDERLYING RANGE: 1 TO 6

SPFTPTX
JF-4. IF JF-1 SPLSTWK= 1 AND JF-3 SPNUMJOB = 1, ASK:
Does she work part time or full time, or some of each? By full time I mean 35 or more hours a week.

ELSE IF JF-1 SPLSTWK= 1 AND (JF-2 SPNUMJOB > 1 OR JF-3 SPNUMJOB = DK/RF), ASK:
At her primary job, does she work part time or full time, or some of each? By full time I mean 35 or more hours a week.

ELSE IF (JF-1 SPLSTWK = 2 OR 3 OR JF-2 SPPAYJOB = 1) AND SJF-3 PNUMJOB = 1, ASK:
Please think about the last week she worked on her job. Did she work part time or full time, or some of each? By full time I mean 35 or more hours a week.

ELSE IF (JF-1 SPLSTWK = 2 OR 3 OR JF-2 SPPAYJOB = 1) AND (JF-3 SPNUMJOB > 1 OR JF-3 SPNUMJOB = DK/RF), ASK:

Please think about the last week she worked on her primary job. Did she work part time or full time, or some of each? By full time I mean 35 or more hours a week.

Full-time..............1
Part time..............2
Some of each...........3

FLOW CHECK J-24: COMPUTE SPEARNTY (WHETHER R'S SPOUSE/PARTNER EVER WORKED).

SET SPEARNTY = NULL/BLANK.
IF MARSTAT NE 1 OR 2 THEN SPEARNTY = NULL/BLANK.
ELSE IF SPWRKST = 1 (YES) OR SPPAYJOB = 1 (YES) THEN SPEARNTY = 1 (YES).
ELSE SPEARNTY = 2 (NO).

Attitudes Towards Sex, Contraception, Marriage, Gender and Parenthood (JG-JH)

{ ASKED FOR ALL
JGINTRO1
JG-0. Please look at Card 84. Next, I would like to get your opinion on some matters concerning family life. I will read you some statements, and I would like you to tell me if you strongly agree, agree, disagree, or strongly disagree. The first is:

◆*ENTER [1] to continue*

[SHOW CARD 84]

STAYTOG
JG-2. Divorce is usually the best solution when a couple can't seem to work out their marriage problems.

◆*PROBE (WC) if R says, "Neither agree nor disagree"*

[SHOW CARD 84]

Strongly agree 1
Agree 2
Disagree 3
Strongly disagree..............................4
If R insists: Neither agree nor disagree 5

SAMESEX
JG-3. Sexual relations between two adults of the same sex are all right.

◆*PROBE (WC) if R says, "Neither agree nor disagree"*

[SHOW CARD 84]

Strongly agree 1
Agree 2
Disagree 3
Strongly disagree..............................4
If R insists: Neither agree nor disagree 5

SXOK18
JG-5. It is all right for unmarried 18 year olds to have sexual intercourse if they have strong affection for each other.

◆*PROBE (WC) if R says, "Neither agree nor disagree"*

[SHOW CARD 84]

Strongly agree 1
Agree 2
Disagree 3
Strongly disagree................................4
If R insists: Neither agree nor disagree 5

SXOK16
JG-6. It is all right for unmarried 16 year olds to have sexual intercourse if they have strong affection for each other.

◆*PROBE (WC) if R says, "Neither agree nor disagree"*

[SHOW CARD 84]

Strongly agree 1
Agree 2
Disagree 3
Strongly disagree................................4
If R insists: Neither agree nor disagree 5

CHUNLESS
JG-6a. People can't be really happy unless they have children.

◆*PROBE (WC) if R says, "Neither agree nor disagree"*

[SHOW CARD 84]

Strongly agree 1
Agree 2
Disagree 3
Strongly disagree................................4
If R insists: Neither agree nor disagree 5

CHSUPPOR
JG-8. It is okay for a young, unmarried woman to have and raise a child.

◆*PROBE (WC) if R says, "Neither agree nor disagree"*

[SHOW CARD 84]

Strongly agree 1
Agree 2
Disagree 3
Strongly disagree................................4
If R insists: Neither agree nor disagree 5

GAYADOPT
JG-9. Gay or lesbian adults should have the right to adopt children.

◆*PROBE (WC) if R says, "Neither agree nor disagree"*

[SHOW CARD 84]

Strongly agree 1
Agree 2
Disagree 3
Strongly disagree..............................4
If R insists: Neither agree nor disagree 5

OKCOHAB
JG-10. A young couple should not live together unless they are married.

◆*PROBE (WC) if R says, "Neither agree nor disagree"*

[HELP AVAILABLE]

[SHOW CARD 84]

Strongly agree 1
Agree 2
Disagree 3
Strongly disagree..............................4
If R insists: Neither agree nor disagree 5

FLOW CHECK J-25: IF (rstrstat = 0 and (pstrstat = (. or 0)) and, if there is a wife/partner, she is not currently pregnant (AB-1 MARSTAT = (1 or 2) and CH-1 CWPPRGNW NE 1), **ASK JG-14 REACTSLF. ELSE IF rstrstat** NE **0 or (pstrstat** NE **(. or 0)) or (AB-1 MARSTAT = (1 or 2) and CH-1 CWPPRGNW = 1), GO TO FLOW CHECK J-26.**

{ ASKED IF NEITHER THE MAN NOR HIS WIFE/PARTNER, IF ANY, ARE STERILE AND HIS
{ WIFE/PARTNER IS NOT CURRENTLY PREGNANT
REACTSLF
JG-14. IF R IS CURRENTLY MARRIED (AB-1 MARSTAT=1) AND HIS WIFE IS NOT CURRENTLY PREGNANT (CH-1 CWPPRGNW NE 1), ASK:
If you got your wife pregnant now how would you feel? Would you be very upset, a little upset, a little pleased, or very pleased?

ELSE IF R IS CURRENTLY COHABITING (AB-1 MARSTAT=2) AND HIS PARTNER IS NOT CURRENTLY PREGNANT (CH-1 CWPPRGNW NE 1), ASK:
If you got your partner pregnant now how would you feel? Would you be very upset, a little upset, a little pleased, or very pleased?

ELSE IF R IS NOT MARRIED OR COHABITING (AB-1 MARSTAT NE 1 or 2), ASK:
If you got a female pregnant now how would you feel? Would you be very upset, a little upset, a little pleased, or very pleased?

◆*PROBE (WC) if R says "Neither upset nor pleased" or he "wouldn't care."*

Very upset 1
A little upset 2

A little pleased 3
Very pleased 4
If R insists: He wouldn't care....... 5

FLOW CHECK J-26: IF ANYKIDS = 1 (YES), GO TO JG-16 MARRFAIL.
(EVER HAD BIOLOGICAL OR ADOPTED CHILDREN)

{ ASKED IF R DOES NOT HAVE ANY BIOLOGICAL OR ADOPTED CHILDREN
CHBOTHER
JG-15. If it turns out that you do not have any children, would that bother you a great deal, some, a little, or not at all?

[HELP AVAILABLE]

A great deal 1
Some 2
A little 3
Not at all 4

{ ASKED OF ALL
MARRFAIL
JG-16. IF R is sterile (rstrstat NE 0) or, if R has a wife /partner, and she is sterile or currently pregnant (AB-1 MARSTAT = (1 OR 2) and (pstrstat NE (. or 0) or currpreg = 1)) [JG-14 REACTSLF not asked]) AND
If he has children (ANYKIDS = 1 (YES) [IH-15 CHBOTHER not asked]), SAY: Marriage has not worked out for most people I know.

ELSE IF he is not sterile (rstrstat = 0) and, if he has a wife/partner, she is not sterile nor pregnant (AB-1 MARSTAT = (1 or 2) and (pstrstat = (. or 0) and currpreg NE 1)) [IH-14 REACTSLF asked]) OR
If R doesn't have children (ANYKIDS = 1) [JG-15 CHBOTHER asked]), SAY: Please look again at Card 84 and tell me if you agree or disagree with these statements. Marriage has not worked out for most people I know.

◆ *PROBE (WC) if R says, "*Neither agree nor disagree"

[SHOW CARD 84]

Strongly agree 1
Agree 2
Disagree 3
Strongly disagree..............................4
If R insists: Neither agree nor disagree 5

CHCOHAB
JG-17. It is okay to have and raise children when the parents are living together but not married.

◆ *PROBE (WC) if R says, "*Neither agree nor disagree"

[SHOW CARD 84]

Strongly agree 1
Agree 2
Disagree 3
Strongly disagree..............................4

If R insists: Neither agree nor disagree 5

PRVNTDIV
JG-18. Living together before marriage may help prevent divorce.

◆ *PROBE (WC) if R says, "*Neither agree nor disagree*"*

[SHOW CARD 84]

Strongly agree 1
Agree 2
Disagree 3
Strongly disagree...............................4
If R insists: Neither agree nor disagree 5

SEXNEEDS
JG-19a. Men have greater sexual needs than women.

◆ *PROBE (WC) if R says, "Neither agree nor disagree"*

[SHOW CARD 84]

Strongly agree 1
Agree 2
Disagree 3
Strongly disagree...............................4
If R insists: Neither agree nor disagree 5

WHENSICK
JG-19b. Men only need to see a doctor when they are hurt or sick.

◆ *PROBE (WC) if R says, "Neither agree nor disagree"*

[SHOW CARD 84]

Strongly agree 1
Agree 2
Disagree 3
Strongly disagree...............................4
If R insists: Neither agree nor disagree 5

SHOWPAIN
JG-19c. When a man is feeling pain he should not let it show.

◆ *PROBE (WC) if R says, "Neither agree nor disagree"*

[SHOW CARD 84]

Strongly agree 1
Agree 2
Disagree 3
Strongly disagree...............................4
If R insists: Neither agree nor disagree 5

FLOW CHECK J-26b: COMPUTE PMARCOHB (WHETHER R EVER COHABITED PREMARITALLY):

IF R HAS NEVER MARRIED (FMARIT=0 or 5) THEN SET PMARCOHB=sysmis (inapplicable).

ELSE IF R HAS EVER MARRIED (FMARIT=1,2,3,4) THEN DO:

IF ANY "YES" RESPONSE ON THE FOLLOWING 15 VARIABLES FOR PREMARITAL COHABITATION (CA-4 LIVTOGWF, DB-3 LIVTOGN, LIVTOGN2-LIVTOGN3, or EB-4 LIVTOGN4-LIVTOG14, SET PMARCOHB=1 (yes).

ELSE SET PMARCOHB=5 (no).

IF R IS CURRENTLY MARRIED (AB-1 MARSTAT = 1), GO TO FLOW CHECK J-27.
ELSE IF R IS CURRENTLY COHABITING (AB-1 MARSTAT = 2), GO TO JG-21 MARRCHANCE.
ELSE ASK JG-20 COHCHANCE.

{ ASKED IF R IS NOT CURRENTLY MARRIED OR COHABITING
COHCHANCE
JG-20. IF PMARCOHB = YES OR EVRCOHAB = YES, ASK:
Please look at Card 58. Do you think that you will ever again live together with a woman to whom you are not married?

ELSE IF PMARCOHB = (NO or BLANK) AND EVRCOHAB = NO, ASK:
Please look at Card 58. Do you think that you will ever live together with a woman to whom you are not married?

♦ *If R insists he does not know, enter [Ctrl] + [D]*

[SHOW CARD 58]

Definitely yes 1
Probably yes 2
Probably no 3
Definitely no 4

{ ASKED IF R IS NOT CURRENTLY MARRIED
MARRCHANCE
JG-21. IF FMARIT in (2, 3, 4) (previously married) and COHCHANCE = . (blank/sysmis), ASK:
Please look at Card 58. You may have already told me this, but do you think that you will get married again someday?

ELSE IF FMARIT in (2, 3, 4) (previously married) and COHCHANCE NE . (blank/sysmis), ASK:
You may have already told me this, but do you think that you will get married again someday?

ELSE IF FMARIT = 0 or 5 (never married and **COHCHANCE = . (blank/sysmis)**, ASK::
Please look at Card 58. You may have already told me this, but do you think that you will get married someday?

ELSE IF FMARIT = 0 or 5 (never married and **COHCHANCE NE. (blank/sysmis)**, ASK::
You may have already told me this, but do you think that you will get married someday?

♦ *If R insists he does not know, enter [Ctrl] + [D]*

[SHOW CARD 58]

Definitely yes 1
Probably yes 2
Probably no 3
Definitely no 4

FLOW CHECK J-26c: IF R GIVES A RESPONSE OTHER THAN "DEFINITELY NO" or DK/RF ON JG-21 MARRCHANCE (MARRCHANCE = 1, 2, 3), ASK JG-22 PMARCOH.
ELSE, IF MARRCHANCE = 4, DK/RF, GO TO JHINT1 JH-1.

{ ASKED IF R SAYS HE MAY (RE)MARRY SOMEDAY
PMARCOH
JG-22. Again, y**ou may have already told me this, but do** you think that you will live together with your future wife before getting married?

♦ *If R insists he does not know, enter [Ctrl] + [D]*

[SHOW CARD 58]

Definitely yes 1
Probably yes 2
Probably no 3
Definitely no 4

Attitudes Towards Condoms (JH)

FLOW CHECK J-27: IF AGESCRN LT 25, ASK JH-1 JHINT1.
ELSE IF AGESCRN GE 25, GO TO JH-6 ACASILANG.

{ ASKED ONLY IF R AGED 15-24 YEARS
LESSPLSR
JH-2. IF RHADSEX = 1, SAY:
The next question is about what might happen the next time you had sex and you used a condom.

ELSE IF RHADSEX = (0 OR 2), SAY:
The next question is about what might happen if you had sex and you used a condom.

Please look at Card 21. What is the chance that if you used a condom during sex, you would feel less physical pleasure?

[SHOW CARD 21]

No chance.........................1
A little chance...................2
50-50 chance 3
A pretty good chance...............4
An almost certain chance...........5

{ ASKED ONLY IF R AGED 15-24 YEARS
EMBARRAS
JH-4. IF RHADSEX = 1, SAY: Now imagine that you are having sex for the first time with a new partner. What is the chance that it would be embarrassing for you and a new partner to discuss using a

condom?

ELSE IF RHADSEX = (0 OR 2), SAY: What is the chance that it would be embarrassing for you and a partner to discuss using a condom?

[SHOW CARD 21]

No chance..........................1
A little chance....................2
50-50 chance 3
A pretty good chance...............4
An almost certain chance...........5

{ INTERVIEWER ANSWERS FOR ALL
ACASILANG

JH-6. ☑ *Interviewer Checkpoint*

- *Should ACASI be conducted in English or Spanish?*

English............................1
Spanish............................2

UNDERLYING ATTRIBUTES: NODK, NORF, NOEMPTY
