# Supplementary material for: Nationally representative prevalence estimates of gay, bisexual, and other men who have sex with men who have served in the U.S. military
Source: PLoS One. 2017 Aug 1;12(8):e0182222. doi: 10.1371/journal.pone.0182222 (PMC5538666; doi:10.1371/journal.pone.0182222)
Supplement: S2 File — (PDF) [file pone.0182222.s002.pdf]

SECTION K

Audio CASI

VARIABLES IMPORTED FROM EARLIER SECTIONS:

RSTATE	USED FOR TANF FILL IN PUBASST KL-4 AND PUBASTYP KL-5 (FROM PRELOAD)
LASTYEAR_FILL	YEAR FILL FOR YEAR OF INTERVIEW - 1 YR ("2005") (FC A-1)
AGE_R	R'S CURRENT AGE (FROM A)
AGESCRN	R'S AGE AT SCREENER (FROM A)
MARSTAT:	MARITAL STATUS (FROM A)
CMLSTYR_FILL	MO/YR FILL FOR 12 MOS BEFORE INTERVIEW (FROM A)
WOMREL	RELATIONSHIP OF WOMAN IN HH TO R (FROM A)
ROSCNT	NUMBER OF PEOPLE IN HH (INCLUDING RESPONDENT) (FROM A)
EVRMARRY	WHETHER R HAS EVER BEEN MARRIED (FROM A)
EVRCOHAB	WHETHER R HAS EVER COHABITED WITH A NONMARITAL PARTNER (FROM A)
P1NAME_FILL	NAME/INITIALS OF MOST RECENT SEXUAL PARTNER (FROM B)
P2NAME_FILL	NAME/INITIALS OF 2ND MOST RECENT SEXUAL PARTNER (FROM B)
P3NAME_FILL	NAME/INITIALS OF 3RD MOST RECENT SEXUAL PARTNER (FROM B)
CMLSXP1_FILL:	FILL FOR DATE OF LAST SEX WITH LAST PARTNER (FROM FC B-19)
CMLSXP2_FILL:	FILL FOR DATE OF LAST SEX WITH 2ND-TO-LAST PARTNER (FROM FC B-27)
CMLSXP3_FILL:	FILL FOR DATE OF LAST SEX WITH 3RD-TO-PARTNER (FROM FC B-35)
CURRPRTS	# OF CURRENT, NONMARITAL, NONCOHABITING PARTNERS (FROM D)
PXCURR[1]	WHETHER P1 IS A CURRENT PARTNER (FROM D)
PXCURR[2]	WHETHER P2 IS A CURRENT PARTNER (FROM D)
PXCURR[3]	WHETHER P3 IS A CURRENT PARTNER (FROM D)
TOTPREGS_C	TOTAL # OF PREGNANCIES COLLECTED THROUGHOUT SECTIONS C-F OF INTERVIEW, INCLUDING CURRENT PREGS (FROM F)
TOTPREGS_R	TOTAL # OF PREGNANCIES R REPORTED IN DIRECT QUESTION, INCLUDING CURRENT PREGS (FROM F)
RWRKST	R WORK STATUS (BASED ON CURRENT STATUS QUESTION)(FROM FC J-10)
REARNTY	WHETHER R IS CURRENTLY WORKING, OR EVER WORKED AT ALL (FROM FC J-13)
ACASILANG	LANGUAGE FOR ADMINISTERING ACASI (FROM J)

VARIABLES CREATED IN THIS SECTION & OUTPUT TO DATA FILE:

PREGACASI	NUMBER OF PREGNANCIES REPORTED IN ACASI (FLOW CHECK K-4B)
ANYORAL	WHETHER R HAS EVER HAD ORAL SEX WITH A FEMALE (FC K-6C)
OPPSEXANY	WHETHER R HAS HAD VAGINAL, ORAL, OR ANAL SEX WITH A FEMALE (FC K-7)
OPPSEXGEN	WHETHER R HAS HAD MALE-GENITAL-INVOLVING SEX WITH A FEMALE (I.E., DOESN'T INCLUDE FEMTOUCH) (FLOW CHECK K-7)
OPPLIFENUM	NUMBER OF OPPOSITE-SEX PARTNERS IN LIFETIME FOR ALL TYPES OF SEX (FC K-9)
OPPYEARNUM	NUMBER OF OPPOSITE-SEX PARTNERS IN LAST 12 MOS FOR ALL TYPES OF SEX (FC K-9f)
SAMESEXANY	WHETHER R HAS HAD ORAL OR ANAL SEX WITH A MALE (FLOW CHECK K-13A)
SAMLIFENUM	NUMBER OF SAME-SEX PARTNERS IN LIFETIME (FC K-14d)
SAMYEARNUM	NUMBER OF SAME-SEX PARTNERS IN LAST 12 MOS (FC K-14f)

VARIABLES CREATED IN THIS SECTION & ARE NOT OUTPUT TO DATA FILE:

POVTHRHLD_FILL	INCOME FILL USED FOR POVERTY RATIO CALCULATION (FC K-19a)
TANF_FILL	STATE TANF PROGRAM FILL USED IN PUBASST KL-4 & PUBASTYP KL-5 (FC K-20)
SNAP_FILL	STATE SNAP PROGRAM CARD NAME FILL USED IN FOODSTMP KL-6 (FC K-21)

{ Read by interviewer from the screen.

INTRO_K1

KA-0a. For this last part of the interview, I'll turn the computer over to you so that you can enter your answers yourself. We have these headphones so that you can listen to the questions in privacy, and you can also read the questions on the computer screen. I will not be able to hear the questions or see the answers you type into the computer. After I explain a few of the keys that you'll be using, I'll help you with the first few practice questions, just to get you started. Then I'll leave you on your own to answer the rest of the questions in privacy.

When you are done with this section, a screen will come up that will tell you how to lock away your responses so that no one can see how you answered the questions. Then you can return the computer to me.

- *ENTER [1] to continue.*

INTRO_K1b
KA-0b. ☑ *Interviewer Checkpoint*

- *Explain the following things to R:*

Connect the headphones to the laptop.
Give the computer to Respondent.
Show Respondent where to find number keys, [Enter], [Backspace], [F11], [F12], and Hyphen keys.

Show Respondent the Aid Card.
Explain how to adjust the volume.

Explain that you will be doing an unrelated task while Respondent completes Audio CASI, but Respondent should feel free to interrupt with questions.

The next screen is for the Respondent.

- *ENTER [1] to continue*

<u>A-CASI PRACTICE QUESTIONS (KA)</u>

{ Machine Audio begins here.
INTRO_K2
KA-0c. These questions are for you to practice with. The interviewer is going to help you do this.

You may press the [BACKSPACE] key to clear an entry when you want to change an answer, or when the computer asks you to correct an answer.

Please press the large [Enter] key on the right side of the keyboard to see the first question.

TYPE: STRING [1]; ATTRIBUTES: EMPTY ALLOWED

FLOW CHECK K-0a: Create an array to loop through PRACYEAR[X] and PRACMNTH[X] up to 2 times.

PRACYEAR[X]
KA-1. In what year were you born?

Please enter the 4-digit year you were born and press the [Enter] key.

UNDERLYING RANGE: 1966 to 2005

PRACMNTH[X]
KA-2. In what month in [PRACYEAR] were you born?

Please enter the number for the month.

January 01

February 02
March 03
April 04
May 05
June 06
July 07
August 08
September 09
October 10
November 11
December 12

PRACCNFM[X]
KA-3. The computer has recorded that you were born in [PRACMNTH, PRACYEAR]. Is this correct?

YES 1 (KA-0 INTROK3a)
NO ....5 (FLOW CHECK K-1)

FLOW CHECK K-1: **IF PRACCNFM=5 AND PRACYEAR[1] NE EMPTY PRACYEAR KA-1. ELSE IF PRACCNFM NE 5 OR PRACYEAR[2] = RESPONSE AND PRACMNTH[2] = RESPONSE 5, GO TO INTROK3a.**

INTROK3a
KA-3a. Thank you. Now we will go over a few keystrokes which will help you complete the survey.

Please press [Enter] to continue.

TYPE: STRING [1]; ATTRIBUTES: EMPTY ALLOWED

INTROK3ab
KA-3ab. If you want to replay the audio, press the [F11] key. It is located near the top right side of the keyboard.

Please press [Enter] to continue.

TYPE: STRING [1]; ATTRIBUTES: EMPTY ALLOWED

INTROK3b
KA-3b. If you want to hide the question, press the [F12] key. To make the question reappear, simply press [F12] again. The [F12] key is located near the [F11] key on the top right side of the keyboard.

Please press [Enter] to continue.

TYPE: STRING [1]; ATTRIBUTES: EMPTY ALLOWED

INTROK3c
KA-3c. If you do not know the answer to a question, press the [CTRL] and [D] keys at the same time.

The [CTRL] key is at the bottom left of the keyboard. It is labeled “Ctrl”.

Please press [Enter] to continue.

TYPE: STRING [1]; ATTRIBUTES: EMPTY ALLOWED

INTROK3d
KA-3d. If you do not wish to answer a particular question, press the [CTRL] and [R] keys at the same time.

Please press [Enter] to continue.

TYPE: STRING [1]; ATTRIBUTES: EMPTY ALLOWED

INTROK3e
KA-3e. If you have any questions about how to use the computer, please ask your interviewer now. Otherwise, please press the [Enter] key to continue on your own.

TYPE: STRING [1]; ATTRIBUTES: EMPTY ALLOWED

INTRO_K4
INTRO-K4. These first questions are about your general health.

Please press [Enter] to continue.

TYPE: STRING [1]; ATTRIBUTES: EMPTY ALLOWED

GENHEALT
KA-4. In general, how is your health? Would you say it is...

Excellent 1
Very good 2
Good 3
Fair 4
Poor 5

RHEIGHT_FT
KA-5. How tall are you?
First, please select the number of feet, then press [Enter].

3 feet 3
4 feet 4
5 feet 5
6 feet 6
7 feet 7

{ RHEIGHT_FT<>DK AND RHEIGHT_FT <>rf
RHEIGHT_IN
KA-5. Now please select the number of inches and then press [Enter].

0 inches 00
1 inch 01
2 inches 02
3 inches 03
4 inches 04
5 inches 05
6 inches 06
7 inches 07
8 inches 08
9 inches 09
10 inches 10
11 inches 11

RWEIGHT
KA-6. How much do you weigh?

Please answer in pounds and then press [Enter].

UNDERLYING RANGE: 50 to 995

Significant Events (KB)

INTRO_K5
KB-0. The next few questions are about some things that you may have experienced recently in your life. We know that some of these questions are about things that you may not think about or talk about often. These things may be difficult to remember and some are personal.

Because this information is very important, please take as much time as you need to read the questions and put your answers into the computer in complete privacy. Your interviewer will never know how you answer and will not ask you any questions about your answers.

Please press [Enter] to continue.

TYPE: STRING [1]; ATTRIBUTES: EMPTY ALLOWED

SHELTER
KB-1. In the last 12 months, that is, since (CMLSTYR_FILL) have you stayed overnight in a shelter for the homeless or some other type of shelter?

Yes 1
No 5

JAILED
KB-2. In the last 12 months, have you spent any time in a jail, prison or a juvenile detention facility?

Yes 1
No 5

FLOW CHECK K-2: IF KB-2 JAILED = NO, DK, OR RF, ASK KB-3 JAILED2.
ELSE IF KB-2 JAILED = YES, GO TO FLOW CHECK K-2A.

JAILED2
KB-3. Have you ever spent time in a jail, prison or juvenile detention center?

Yes 1
No 5

FLOW CHECK K-2a: IF KB-2 JAILED = NO, DK, OR RF AND KB-3 JAILED2 = NO, DK, OR RF, THEN GO TO FLOW CHECK K-2B. ELSE ASK KB-4 FRQJAIL.

{ Asked if ever been in jail (JAILED=1 or JAILED2=1)
FRQJAIL
KB-4. Have you been in jail, prison, or a juvenile detention facility only one time or more than one time?

Only one time...................1
Or more than one time...........2

/ "Length of time R was jailed or detained the last time"
FRQJAIL2
KB-5. If KB-4 FRQJAIL = 1, ask:
How long were you in jail, prison, or juvenile detention?

Else if KB-4 FRQJAIL = 2, DK, OR RF, ask:
The last time you were in jail, prison, or juvenile detention, how long were you in?

One month or less...1
More than one month but less than one year.................2
One year..3
More than one year 4

FLOW CHECK K-2B: IF AGESCRN GE 25, GO TO INTRO_K6 (KC-0).

{ Asked only if R is 15-24 years old
EVSUSPEN
KB-6. Have you ever been suspended or expelled from school?

Yes 1
No ....5 [**GO TO INTRO_K6 (KC-0)**]

{ Asked only if R is 15-24 years old
GRADSUSP
KB-7. What grade were you in when you were suspended or expelled from school? If you were suspended or expelled more than once, please enter the grade you were in the most recent time.

UNDERLYING RANGE: 1..12

Substance Use (KC)

INTRO_K6
KC-0. These next questions are about your use of alcohol and other substances.

Please press [Enter] to continue.

TYPE: STRING [1]; ATTRIBUTES: EMPTY ALLOWED

DRINK12
KC-1. During the last 12 months, that is, since (CMLSTYR_FILL), how often have you had beer, wine, liquor, or other alcoholic beverages?

Never 1
Once or twice during the year 2
Several times during the year 3
About once a month 4
About once a week 5
About once a day 6

FLOW CHECK K-3: IF KC-1 DRINK12 = 2,3,4,5,6, OR DK, THEN ASK KC-1a_U UNIT30D. ELSE IF DRINK12 = 1 OR RF, GO TO KC-3 POT12.

UNIT30D
KC-1a_U. This next question asks about your drinking over the past 30 days. Would you prefer to answer in terms of days per week or days per month?

Days per week 1
Days per month 5

IF RF, GO TO KC-2 BINGE12

DRINK30D

KC-1a_N. IF UNIT30D = 1, ASK:
During the past 30 days, that is, since (mo/day/yr), on how many days per week did you have at least one drink of any alcoholic beverage such as beer, wine, a malt beverage or liquor?

ELSE IF UNIT30D = 5 OR DK, ASK:
During the past 30 days, that is, since (mo/day/yr), on how many days did you have at least one drink of any alcoholic beverage such as beer, wine, a malt beverage or liquor?

UNDERLYING RANGE: 0 to 30

FLOW CHECK K-3a: IF KC-1a_N DRINK30D = 0, GO TO KC-2 BINGE12.
ELSE IF DRINK30D = DK OR RF, GO TO KC-1c BINGE30.
ELSE IF 0 LE DRINK30D LE 30, ASK KC-1n DRINKDAY.

DRINKDAY

KC-1b. One drink is equivalent to a 12-ounce beer, a 5-ounce glass of wine, or a drink with one shot of liquor. During the past 30 days, on the days when you drank, about how many drinks did you drink on the average?

NOTE: A 40 ounce beer would count as 3 drinks, or a cocktail drink with 2 shots would count as 2 drinks.

UNDERLYING RANGE: 1 to 95

BINGE30

KC-1c. Considering all types of alcoholic beverages, how many times during the past 30 days did you have 5 or more drinks on an occasion?

UNDERLYING RANGE: 0 to 95

DRNKMOST

KC-1d. During the past 30 days, what is the largest number of drinks you had on any occasion?

UNDERLYING RANGE: 1 to 95

BINGE12

KC-2. During the last 12 months, that is, since CMLSTYR_FILL), how often did you have 5 or more drinks within a couple of hours?

Never 1
Once or twice during the year 2
Several times during the year 3
About once a month 4
About once a week 5
About once a day 6

POT12

KC-3. During the last 12 months, how often have you smoked marijuana?

Never 1
Once or twice during the year 2
Several times during the year 3
About once a month 4
About once a week 5
About once a day or more 6

COC12
KC-4. During the last 12 months, how often have you used cocaine?

Never 1
Once or twice during the year 2
Several times during the year 3
About once a month or more 4

CRACK12
KC-5. During the last 12 months, how often have you used crack?

Never 1
Once or twice during the year 2
Several times during the year 3
About once a month or more 4

CRYSTMTH12
KC-5a.During the last 12 months, how often have you used Crystal or meth, also known as tina, crank, or ice?

Never 1
Once or twice during the year 2
Several times during the year 3
About once a month or more 4

INJECT12
KC-6. During the last 12 months, how often have you shot up or injected drugs other than those prescribed for you? By shooting up, we mean anytime you might have used drugs with a needle, by mainlining, skin-popping, or muscling.

Never 1
Once or twice during the year 2
Several times during the year 3
About once a month or more 4

Pregnancy/Abortion (KD)

INTRO_K7
KD-0. Here are a few questions asking about pregnancies you may have fathered. Sometimes men who take part in the study are reluctant to tell an interviewer about their experience with pregnancies, especially if the pregnancies ended in abortion or with children they no longer live with.

Please press [Enter] to continue.

TYPE: STRING [1]; ATTRIBUTES: EMPTY ALLOWED

FLOW CHECK K-4: **IF R HAS NOT REPORTED FATHERING ANY PREGNANCIES IN SECTIONS C-F OF THE QUESTIONNAIRE (TOTPREGS_R = 0 AND TOTPREGS_C = 0), ASK KD-1 MADEPREG.**
ELSE IF TOTPREGS_R > 0 OR TOTPREGS_C > 0, GO TO KD-2b PREGTOT2.

MADEPREG
KD-1. To the best of your knowledge, have you ever made someone pregnant?

Yes 1

No ....5 (FLOW CHECK K-5)

PREGTOT2
KD-2. To the best of your knowledge, how many times have you ever made someone pregnant? Please include any pregnancies you may have already told the interviewer about.

UNDERLYING RANGE: 1 to 95

FLOW CHECK K-4b: CREATE PREGACASI (NUMBER OF PREGNANCIES REPORTED IN ACASI)

IF KD-1 MADEPREG = 5, DK, or RF, SET PREGACASI=0.
ELSE PREGACASI = KD-2 PREGTOT2.

NUMABORT
KD-3. To the best of your knowledge, how many of these pregnancies ended in abortion?

UNDERLYING RANGE: 0 to 95

NUMLIVEB
KD-4. ASK ONLY IF KD-3 NUMABORT < KD-2 PREGACASI:
To the best of your knowledge, how many of these pregnancies resulted in a baby being born?

Twins or triplets from a pregnancy count as one pregnancy.

UNDERLYING RANGE: 0 to 95

FLOW CHECK K-5: IF AGESCRN GE 25, GO TO INTRO_K8.

TOLDPREG
KD-5. Have you ever been told by someone that you may have made her pregnant?

Yes 1
No ....5 (INTRO_K8)

WHATHAPP
KD-6. The last time you were told by someone that you may have made her pregnant, ...

Did it turn out that she was pregnant and you were the father, ...1
Or was she pregnant but you were not the father, ...2
Or did it turn out that she was not pregnant? 3

Sex with Females (KE)

INTRO_K8
KE-0. The next questions are about sexual experiences that you may have had with a female.

Please press [Enter] to continue.

TYPE: STRING [1]; ATTRIBUTES: EMPTY ALLOWED

FLOW CHECK K-6: IF AGESCRN LT 20 AND IF R HAS NEVER BEEN MARRIED AND NEVER COHABITED (EVRMARRY=0 AND EVRCOHAB=0), READ INTRO-K9a.

ELSE IF AGESCRN GE 20 OR IF R HAS EVER BEEN MARRIED OR COHABITED (EVRMARRY=1 OR EVRCOHAB=1), GO TO INTRO-K9b.

{AGESCRN LT 20 AND EVRMARRY=0 AND EVRCOHAB=0
INTRO_K9a
KE-0a. Here are some things you may have done with a female. If you have ever done this at least one time with a female, answer yes. If you have never done this, answer no.

Please press [Enter] to continue.

TYPE: STRING [1]; ATTRIBUTES: EMPTY ALLOWED

{AGESCRN LT 20 AND EVRMARRY=0 AND EVRCOHAB=0
FEMTOUCH
KE-1. Has a female ever touched your penis until you ejaculated, or "came"?

Yes 1
No 5

ONCE FEMTOUCH IS ANSWERED GO TO FC K-6B

{AGESCRN GE 20 OR EVRMARRY=1 OR EVRCOHAB=1
INTRO_K9b
KE-1b. Here are some things you may have done with a female. If you have ever done this at least one time with a female, answer yes. If you have never done this, answer no.

Please press [Enter] to continue.

TYPE: STRING [1]; ATTRIBUTES: EMPTY ALLOWED

FLOW CHECK K-6B: IF EVRMARRY=1 OR EVRCOHAB=1 OR
(0 < KD-2 PREGACASI <= 95) OR
(0 < TOTPREGS_C < 97) OR
(0 < TOTPREGS_R <= 95),

THEN GO TO KE-3 CONDVAG.

ELSE, ASK KE-2 VAGSEX.

VAGSEX
KE-2. Have you ever put your penis in a female's vagina (also known as vaginal intercourse)?

Yes 1
No ....5 (KE-5 GETORALF)

AGEVAGR
KE-2b. The first time this occurred, how old were you?

UNDERLYING RANGE: 0 to 45

CONDVAG
KE-3. Did you use a condom the last time you had vaginal intercourse with a female?

Yes 1
No ....5 (KE-5 GETORALF)

WHYCONDL
KE-4. The last time you had vaginal intercourse with a female, did you use

the condom...

To prevent pregnancy, ...1
To prevent diseases like syphilis, gonorrhea or AIDS, ..2
For both reasons, ...3
Or for some other reason 4

GETORALF
KE-5. The next few questions are about oral sex. By oral sex, we mean stimulating the genitals with the mouth. Has a female ever performed oral sex on you, that is, stimulated your penis with her mouth?

Yes 1
No ....5 (KE-7 GIVORALF)

CONDFELL
KE-6. Did you use a condom the last time a female performed oral sex on you?

Yes 1
No 5

GIVORALF
KE-7. Have you ever performed oral sex on a female?

Yes 1
No 5

FLOW CHECK K-6C: COMPUTE ANYORAL:
whether R has ever had oral sex (either type)

SET ANYORAL=INAPP/BLANK SO THAT DK/RF RESPONSES WILL NOT BE ASSUMED TO BE “NO.”
IF KE-5 GETORALF=1 OR KE-7 GIVORALM=1 THEN ANYORAL=1 (YES).

ELSE IF GETORALF=5 AND GIVORALF=5 THEN ANYORAL=5 (NO).

IF ANYORAL=1 AND (KE-2 VAGSEX= 1 OR SYSMIS) AND AGESCRN < 25, ASK KE-7B TIMING.

ELSE GO TO KE-8 ANALSEX.

TIMING
KE-7b.Thinking back to when you had oral sex with a female for the first time, was it before, after, or on the same occasion as your first vaginal intercourse with a female?

Before first vaginal intercourse 1
After first vaginal intercourse 3
Same occasion..........................5

ANALSEX
KE-8. Have you ever put your penis in a female's anus or butt (also known as anal sex)?

Yes 1
No ....5 (FLOW CHECK K-7)

CONDANAL
KE-9. Did you use a condom the last time you had anal sex with a female?

Yes 1

No 5

FLOW CHECK K-7: **COMPUTE OPPSEXANY:**
Whether R has ever had vaginal, oral, or anal sex with a female partner

INITIALIZE OPPSEXANY=null/blank

IF KE-2 VAGSEX=blank THEN OPPSEXANY = 1.
ELSE IF KE-2 VAGSEX=1 OR KE-7 GIVORALF=1 OR KE-5 GETORALF=1 OR KE-8 ANALSEX=1 OR KE-1 FEMTOUCH=1
THEN OPPSEXANY=1 (yes)
ELSE IF VAGSEX=5 AND GIVORALF=5 AND GETORALF=5 AND ANALSEX=5 AND (FEMTOUCH=5 or blank)
THEN OPPSEXANY=5 (NO)

COMPUTE OPPSEXGEN:
Whether R has ever had male-genital-involving sex with a female partner

INITIALIZE OPPSEXGEN=null/blank

IF OPPSEXANY=5 THEN OPPSEXGEN=5.

ELSE IF OPPSEXANY=1 THEN DO:
IF VAGSEX=(1 OR BLANK) OR FEMTOUCH=1 OR ANALSEX=1 OR GETORALF=1
THEN OPPSEXGEN=1;
ELSE OPPSEXGEN=5 (this means that GIVORALF was the only reason that OPPSEXANY=1, so there was no contact with male genitals)

END DO.

IF OPPSEXGEN=1 (yes) AND R HAS REPORTED MORE THAN ONE FORM OF MALE-GENITAL-INVOLVING SEX WITH A FEMALE (MORE THAN 1 “YES” RESPONSE AMONG VAGSEX (or VAGSEX=blank), GETORALF, FEMTOUCH, AND ANALSEX) AND R HAS REPORTED CONDOM USE FOR AT LEAST 1 FORM OF THIS SEXUAL ACTIVITY (AT LEAST 1 “YES” RESPONSE FOR KE-3 CONDVAG, KE-6 CONDFELL, OR KE-9 CONDANAL), ASK KE-10 CONDSEXL.

ELSE IF AGE_R >= 18 GO TO FLOW CHECK K-8.

ELSE IF AGE_R < 18, GO TO FLOW CHECK K-9.

{ ASKED IF R HAS HAD MORE THAN 1 FORM OF SEX INVOLVING MALE GENITALS, AND HE
{ REPORTED USING A CONDOM AT LAST SEX FOR ANY SPECIFIC TYPE

CONDSEXL

KE-10. The very <u>last time</u> you had <u>any type of sex</u> -- that is, vaginal intercourse <u>or</u> anal sex <u>or</u> oral sex -- with a female partner, did you use a condom?

Yes 1
No 5

FLOW CHECK K-7B: **IF AGE_R GE 18, CONTINUE WITH FLOW CHECK K-8.**
ELSE IF AGE_R LT 18, GO TO FLOW CHECK K-9.

Non Voluntary Intercourse: Female - Male (KF)

**FLOW CHECK K-8: IF KE-2 VAGSEX = YES OR BLANK, ASK KF-1 WANTSEX1.
ELSE GO TO KF-2 EVRFORCD.**

WANTSEX1
KF-1. Think back to the very first time you had vaginal intercourse with a female. Which would you say comes closest to describing how much you wanted that first vaginal intercourse to happen?

I really didn’t want it to happen at the time 1
I had mixed feelings -- part of me wanted it to happen at the time and part of me didn’t 2
I really wanted it to happen at the time 3

HOWOLD
KF-1b. How old were you when this first intercourse happened?

UNDERLYING RANGE: 0 to 45

EVRFORCD
KF-2. At any time in your life, have you ever been forced by a female to have vaginal intercourse against your will?

Yes...............1
No................5 (Flow Check K-9)

AGEFORC1
KF-3. How old were you the very first time you were forced by a female to have vaginal intercourse against your will?

UNDERLYING RANGE: 0 to 45

INTROK10
KF-4. The first time this occurred, were any of these kinds of force used?

Please press [Enter] to continue.

TYPE: STRING [1]; ATTRIBUTES: EMPTY ALLOWED

GIVNDRG2
KF-4a. Were you given alcohol or drugs?

Yes.........1
No..........5

SHEBIGOL
KF-4b. Did you do what she said because she was bigger than you or a grown-up, and you were young?

Yes.........1
No..........5

ENDRELA2
KF-4c. Were you told that the relationship would end if you didn’t have sex?

Yes.........1

No..........5

WRDPRES2
KF-4d. Were you pressured into it by her words or actions, but without threats of harm?

Yes.........1
No..........5

THRTPHY2
KF-4e. Were you threatened with physical hurt or injury?

Yes.........1
No..........5

PHYSHRT2
KF-4f. Were you physically hurt or injured?

Yes.........1
No..........5

HELDDWN2
KF-4g. Were you physically held down?

Yes.........1
No..........5

STD/HIV Risking Behaviors: Females (KG)

FLOW CHECK K-9: IF OPPSEXANY=1 (yes), READ INTROK11.
ELSE IF OPPSEXANY=2 (no), GO TO INTROK13.

INTROK11
KG-0. This next section is about your female sex partners. Now please think about any female with whom you have had vaginal intercourse, oral sex, or anal sex -- any of these.

Please press [Enter] to continue.

TYPE: STRING [1]; ATTRIBUTES: EMPTY ALLOWED

FLOW CHECK K-9a: CREATE AN ARRAY TO LOOP THROUGH PARTSLIF[X] UP TO 2 TIMES.

PARTSLIF[X]
KG-1. Thinking about your entire life, how many female sex partners have you had? Please count every partner even those you had sex with only once.

UNDERLYING RANGE: 1 to 995

FLOW CHECK K-9b: IF PARTSLIF[1]= 11, 22, 33, 44, 55, 66, 77, 88, 99, 111, 222, 333, 444,555, 666, 777, or 888, ASK PARTSLFV KG-1v.
ONCE PARTSLFV = NO AND PARTSLIF[2]=RESPONSE, GO TO CHECK K-9d.
ELSE GO TO FLOW CHECK K-9d.

{ASKED ONCE AND FC K-9b IS TRUE
PARTSLFV
KG-1v. It is very important that we get accurate responses to this key

question. The computer recorded that you have had (PARTSLIF[1]) female sex partners in your entire life. If this answer is correct, select "Yes" to move on to the next question. If the computer recorded an incorrect response, please select "No" to change your answer.

Yes 1
No 5

**FLOW CHECK K-9c: IF PARTSLFV = NO, RETURN AND ASK PARTSLIF[2].
ELSE IF PARTSLFV NE NO OR PARTSLIF[2]=RESPONSE, GO TO FLOW CHECK K-9d.**

FLOW CHECK K-9d: DEFINE OPPLIFENUM: ("NUMBER OF OPPOSITE-SEX PARTNERS IN LIFETIME FOR ALL TYPES OF SEX. (computed in FC K-9d)")

**IF KG-1v PARTSLFV = 1 or DK or RF or (PARTSLFV = 5 and KG-1 PARTSLIF_2 = DK or RF), THEN OPPLIFENUM = KG-1 PARTSLIF_1.
ELSE IF PARTSLFV = 5 and PARTSLIF_2 NE DK or RF, THEN OPPLIFENUM = PARTSLIF_2.**

CREATE AN ARRAY TO LOOP THROUGH PARTS12[X] UP TO 2 TIMES.

PARTS12[X]
KG-2. Thinking about the last 12 months, how many female sex partners have you had in the 12 months since (CMLSTYR_FILL)? Please count every partner, even those you had sex with only once in those 12 months.

UNDERLYING RANGE: 0 to 995

**FLOW CHECK K-9b: IF PARTSLIF[1]= 11, 22, 33, 44, 55, 66, 77, 88, 99, 111, 222, 333, 444,555, 666, 777, or 888, ASK PARTSLFV KG-1v.
ONCE PARTSLFV = NO AND PARTSLIF[2]=RESPONSE, GO TO CHECK K-9d.
ELSE GO TO FLOW CHECK K-9d.**

**FLOW CHECK K-9e: IF PARTS12[1] = (11, 22, 33, 44, 55, 66, 77, 88, 99, 111, 222, 333, 444, 555, 666, 777, or 888) AND PARTSLFV HAS NOT BEEN ASKED (R IS ONLY ASKED THIS ACASI ERROR ROUTING QUESTION ONCE), ASK PARTS12V KG-2v.
ONCE PARTS12V NE NO AND PARTS12[2] = RESPONSE, GO TO FLOW CHECK K-9g.
ELSE GO TO FLOW CHECK K-9g.**

**{ASKED ONCE AND FC K-9e IS TRUE AND PARTS12V HAS NOT BEEN ASKED
PARTS12V**
KG-2v. It is very important that we get accurate responses to this key question. The computer recorded that you have had (FEMPRT12[1])female sex partners in the last 12 months. If this answer is correct, select "Yes" to move on to the next question. If the computer recorded an incorrect response, please select "No" to change your answer.

Yes 1
No 5

**FLOW CHECK K-9f: IF PARTS12V = NO, RETURN AND ASK PARTS12[2]
ELSE IF PARTS12V NE NO OR PARTS12[2]=RESPONSE:**

DEFINE OPPYEARNUM: ("NUMBER OF OPPOSITE-SEX PARTNERS IN LAST 12 MONTHS FOR ALL TYPES OF SEX.(computed in FC K-9d)")

IF KG-2v PARTS12V = 1 or DK or RF or (PARTS12V = 5 and KG-2 PARTS12_2 = DK or RF), THEN OPPYEARNUM = KG-2 PARTS12_1.
ELSE IF PARTS12V = 5 and PARTS12_2 NE DK or RF, THEN OPPYEARNUM = PARTS12_2.

THEN GO TO FLOW CHECK K-9g.

FLOW CHECK K-9g: **IF OPPYEARNUM > OPPLIFENUM THEN ASK KG-2YR NEWYEAR. ELSE GO TO FLOW CHECK K-9h.**

NEWYEAR
KG-2YR. Earlier you reported having more female partners in the last 12 months than you have had in your life. One or both of these numbers appear to be entered incorrectly, so those questions will be asked again. Your previous answers are displayed below:

DISPLAY: *[OPPYEARNUM] female partners in last 12 months*

[OPPLIFENUM] female partners in lifetime

How many female partners did you have in the last 12 months?

UNDERLYING RANGE: 0 to 995

NEWLIFE
KG-2LF. How many female partners did you have in your lifetime?

UNDERLYING RANGE: 0 to 995

FLOW CHECK K-9H: **IF KG-2YR NEWYEAR NE SYSMIS (this question was asked), RESET OPPYEARNUM = NEWYEAR.**

IF KG-2LF NEWLIFE NE SYSMIS (this question was asked), RESET OPPLIFENUM = NEWLIFE.

IF R HAS HAD NO FEMALE PARTNERS IN THE LAST 12 MONTHS (OPPYEARNUM = 0), GO TO FLOW CHECK K-10. ELSE, ASK KG-2YRa VAGNUM12.

VAGNUM12
KG-2YRa. ASK IF KE-2 VAGSEX=1 or blank:
Your number of female partners in the last 12 months is displayed below. Thinking of your female partners in the last 12 months, with how many of them did you have vaginal intercourse?

DISPLAY: *[OPPYEARNUM] female partners in last 12 months*

UNDERLYING RANGE: 0 to 995

ORALNUM12
KG-2YRb. ASK IF ANYORAL=1:
(Your number of female partners in the last 12 months is displayed below.) Thinking of your female partners in the last 12 months, with how many of them did you have oral sex, either giving or receiving?

DISPLAY: *[OPPYEARNUM] female partners in last 12 months*

UNDERLYING RANGE: 0 to 995

ANALNUM12
KG-2YRc. ASK IF KE-8 ANALSEX=1:
(Your number of female partners in the last 12 months is displayed below.) Thinking of your female partners in the last 12 months, with how many of them did you have anal sex?

DISPLAY: *[OPPYEARNUM] female partners in last 12 months*

UNDERLYING RANGE: 0 to 995

FLOW CHECK K-10: IF AGE_R < 18 AND CURRPRTS > 0, READ INTROK12.
ELSE (IF AGE_R < 18 AND CURRPRTS = 0) OR IF AGE_R GE 18, GO TO FLOW CHECK K-12.

INTROK12
KG-3. IF CURRPRTS = 1, SAY:
You indicated in the interview that you have 1 current sexual partner. Here are a couple of questions about her.

ELSE IF CURRPRTS = 2 or 3, SAY:
You indicated in the interview that you have (CURRPTR#) current sexual partners. Here are a couple of questions about those partners.

ELSE IF CURRPRTS GT 3, SAY:
You indicated in the interview that you have more than 3 current sexual partners. Here are a couple of questions about some of those partners.

Please press [Enter] to continue.

TYPE: STRING [1]; ATTRIBUTES: EMPTY ALLOWED

FLOW CHECK K-10b: SET CURRCNTR = 1.
ASK CURRPAGE through HOWMUCH (as applicable) for each current partner reported (up to 3).

DISPLAY P1NAME_FILL IF PXCURR[1] = YES.
DISPLAY P2NAME_FILL IF PXCURR[2] = YES.
DISPLAY P3NAME_FILL IF PXCURR[3] = YES.

CURRPAGE
KG-3a. IF ONLY 1 NAME ON SCREEN:
Earlier you reported that you last had sexual intercourse with the person shown on the screen in (CMLSXPX_FILL).

How old was she at that time?

ELSE IF MORE THAN 1 NAME ON SCREEN:
Earlier you reported that you last had sexual intercourse with the (first/second/third) person shown on the screen in (CMLSXPX_FILL).

How old was she at that time?

UNDERLYING RANGE: 5 to 95

FLOW CHECK K-10c: IF KG-3a CURRPAGE = DK, ASK KG-3b RELAGE.
ELSE IF CURRPAGE = RF OR CURRPAGE LE 95, GO TO FLOW CHECK K-11.

RELAGE
KG-3b. Is she older than you, younger than you or about the same age?

Older 1
Younger 2
Same age 3

FLOW CHECK K-10d: IF KG-3b RELAGE = 3, DK, OR RF, GO TO FLOW CHECK K-11.
ELSE IF RELAGE = 1 OR 2, ASK KG-3c HOWMUCH.

HOWMUCH
KG-3c. By how many years?

1-2 years 1
3-5 years 2
6-10 years 3
More than 10 years 4

FLOW CHECK K-11: IF NO MORE CURRENT PARTNERS TO DESCRIBE (counter value exceeds CURRPRTS), GO TO FLOW CHECK K-12. ELSE RETURN TO KG-3a CP#AGE1 TO ASK ABOUT NEXT CURRENT PARTNER.

FLOW CHECK K-12: IF OPPYEARNUM > 0 or OPPYEARNUM = DK, ASK KG-4 NONMONOG.
ELSE IF OPPYEARNUM = 0 OR RF, GO TO INTROK13.

NONMONOG
KG-4. IF AGE_R < 18 AND CURRPRTS > 0, ASK:
Now please think about <u>all</u> of your female sexual partners in the <u>last 12 months</u>, that is since (CMLSTYR_FILL).

In the <u>last 12 months</u>, did you have sex with any females who were also having sex with other people at around the same time?

ELSE IF AGE_R GE 18 OR CURRPRTS = 0, ASK:
In the <u>last 12 months</u>, that is, since (CMLSTYR_FILL), did you have sex with any females who were also having sex with other people at around the same time?

Yes 1
No 5

FLOW CHECK K-12a: IF KG-4 NONMONOG = 5, DK, OR RF, GO TO KG-6 FEMSHT12.

ELSE IF NONMONOG = 1 and 1 < OPPYEARNUM < 995, ASK KG-5a NNONMONOG1.

ELSE IF NONMONOG = 1 and OPPYEARNUM = 1, ASK KG-5b NNONMONOG2. *(if only had 1 female partner in last 12 mos, go right to KG-5b)*

NNONMONOG1
KG-5a. <u>To the best of your knowledge</u>, how many of your female sexual partners in the last 12 months were having sex with other people around the same time?

1 partner 1
2 or more partners......2

**FLOW CHECK K-12b: IF KG-5a NNONMONOG1 = 1, ASK KG-5b NNONMONOG2.
ELSE IF NNONMONOG1 = 2 or DK, ASK KG-5c NNONMONOG3.
ELSE IF NNONMONOG1 = RF, GO TO KG-6 FEMSHT12.**

NNONMONOG2
KG-5b. IF NNONMONOG1=1, ASK:
How many other partners do you think this partner had around the same time as she was having sex with you?

ELSE IF NONMONOG=1 and OPPYEARNUM=1, ASK:
Thinking of your 1 female partner in the last 12 months, how many other partners do you think she had around the same time as she was having sex with you?

1 other partner besides you 1
2 other partners besides you 2
3 or more other partners besides you 3

**FLOW CHECK J-12c: IF KG-4 NONMONOG = 1 and KG-5a NNONMONOG1 = 2, ASK KG-5c NNONMONOG3.
ELSE IF NONMONOG = 1 and NNONMONOG1 = 1, GO TO KG-6 FEMSHT12. *(NNONMONOG1=RF cases were already sent to FEMSHT12.)***

NNONMONOG3
KG-5c. Thinking of your most recent female partner who had other sexual partners, how many other partners do you think she had around the same time as she was having sex with you?

1 other partner besides you 1
2 other partners besides you 2
3 or more other partners besides you 3

FEMSHT12
KG-6. In the last 12 months, that is, since (CMLSTYR_FILL), have you had sex with a female who takes or shoots street drugs using a needle?

Yes 1
No 5

JOHNFREQ
KG-7. In the last 12 months, have you given a female money or drugs in exchange for having sex with you?

Yes 1
No 5

PROSTFRQ
KG-8. In the last 12 months, has a female given you money or drugs to have sex with her?

Yes 1
No 5

HIVFEM12
KG-9. In the last 12 months, have you had sex with a female who you knew was infected with the AIDS virus?

Yes 1
No 5

Sex with Males (KH)

INTROK13
KH-0. The next questions ask about sexual experiences you may have had with another male. Have you ever done any of the following with another male?

Please press [Enter] to continue.

TYPE: STRING [1]; ATTRIBUTES: EMPTY ALLOWED

GIVORALM
KH-1. Have you ever performed oral sex on another male, that is, stimulated his penis with your mouth?

Yes 1
No 5

GETORALM
KH-2. Has another male ever performed oral sex on you, that is, stimulated your penis with his mouth?

Yes 1
No 5

{ Asked if R ever had oral sex with a male partner.
ORALCONDM
KH-2b. ASKED IF KH-1 GIVORALM=1 or KH-2 GETORALM=1:
Did you use a condom the last time you had oral sex with a male?

Yes 1
No 5

ANALSEX2
KH-3. Has another male ever put his penis in your anus or butt (receptive anal sex)?

Yes 1
No 5

{ Asked if R ever had receptive anal sex with a male partner.
ANALCONDM1
KH-3b. ASKED IF KH-3 ANALSEX2=1:
Did you use a condom the last time you had receptive anal sex with a male?

Yes 1
No 5

ANALSEX3
KH-4. Have you ever put your penis in another male’s anus or butt (insertive anal sex)?

Yes 1
No 5

{ Asked if R ever had insertive anal sex with a male partner.
ANALCONDM2
KH-4b. ASKED IF KH-4 ANALSEX3=1:
Did you use a condom the last time you had insertive anal sex

with a male?

Yes 1
No 5

FLOW CHECK K-13A: COMPUTE SAMESEXANY:
Whether R has had oral or anal sex with a male partner

INITIALIZE SAMESEXANY=null/blank
IF GIVORALM=1 or GETORALM=1 or ANALSEX2=1 or ANALSEX3=1,
THEN SAMESEXANY=1 (yes)
ELSE IF GIVORALM=5 and GETORALM=5 and ANALSEX2=5 AND ANALSEX3=5
THEN SAMESEXANY=5 (no).

IF SAMESEXANY = SYSMIS or NO, GO TO FLOW CHECK K-13b.

{ Asked for all who have ever had a male sexual partner (samesexany=1).
MALPRTAGE
KH-5. Thinking of your most recent or last male sex partner, that is, the man with whom you last had oral or anal sex, was he older than you, younger than you, or about the same age?

Older 1
Younger 2
Same age 3

{ Asked for all who have ever had a male sexual partner (samesexany=1).
MALPRTHISP
KH-6. Thinking of this same male partner with whom you last had oral or anal sex, is he Hispanic or Latino, or of Spanish origin?

Yes 1
No 5

{ Asked for all who have ever had a male sexual partner (samesexany=1).
MALPRTRACE
KH-7. Thinking of this same male sexual partner, which of the groups shown below describe his racial background?

Please enter all that apply.

To enter multiple answers, enter the number of the first answer, press the space bar, enter the number of the next answer, and so forth. The space bar is the long key at the bottom of the keyboard, in the middle. Press [Enter] once you're finished entering all your answers.

American Indian or Alaska Native 1
Asian....................................2
Native Hawaiian or Other Pacific Islander..3
Black or African American 4
White 5

<u>Non Voluntary Intercourse: Male -> Male (KI)</u>

FLOW CHECK K-13B: IF AGE_R GE 18, ASK KI-1 EVRFORC2.

ELSE IF AGE_R LT 18 AND IF SAMESEXANY=no or sysmis, GO TO KK-4 ATTRACT.

ELSE IF AGE_R LT 18 AND SAMESEXANY=yes, GO TO INTROK15.

EVRFORC2
KI-1. At any time in your life, have you ever been forced by a male to have oral or anal sex against your will?

Yes.........1
No..........5 (KJ series)

AGEFORC2
KI-2. How old were you the very first time you were forced by a male to have sexual intercourse against your will?

UNDERLYING RANGE: 0 to 45

INTROK14
KI-3. The first time this occurred, were any of these kinds of force used?

Please press [Enter] to continue.

TYPE: STRING [1]; ATTRIBUTES: EMPTY ALLOWED

GIVNDRG3
KI-3a. Were you given alcohol or drugs?

Yes.........1
No..........5

HEBIGOLD
KI-3b. Did you do what he said because he was bigger than you or a grown-up, and you were young?

Yes.........1
No..........5

ENDRELA3
KI-3c. Were you told that the relationship would end if you didn't have sex?

Yes.........1
No..........5

WRDPRES3
KI-3d. Were you pressured into it by his words or actions, but without threats of harm?

Yes.........1
No..........5

THRTPHY3
KI-3e. Were you threatened with physical hurt or injury?

Yes.........1
No..........5

PHYSHRT3
KI-3f. Were you physically hurt or injured?

Yes.........1
No..........5

HELDDWN3
KI-3g. Were you physically held down?

Yes.........1
No..........5

STD/HIV Risking Behaviors: Males (KJ)

FLOW CHECK K-14: IF AGE_R GE 18 AND IF SAMESEXANY = NO, GO TO KK-4 ATTRACT.

INTROK15
KJ-0. This next section is about males with whom you have had sexual contact. Think about any male with whom you have had oral or anal sex.

Please press [Enter] to continue.

TYPE: STRING [1]; ATTRIBUTES: EMPTY ALLOWED

FLOW CHECK K-14a: CREATE AN ARRAY TO LOOP THROUGH MALEPRTS[X] UP TO 2 TIMES.

MALEPRTS[X]
KJ-1. Thinking about your entire life, how many male sex partners have you had?

UNDERLYING RANGE: 1 to 995

FLOW CHECK K-14b: IF MALEPRTS[1] = 11, 22, 33, 44, 55, 66, 77, 88, 99, 111, 222, 333, 444, 555, 666, 777, or 888 ASK MALEPRTSV KJ-1v.
ONCE MALEPRTSV = NO AND MALEPRTS[2]=RESPONSE GOTO CHECK K-14d.
ELSE GOTO FLOW CHECK K-14d.

{ASKED ONCE IF FC K-14b IS TRUE
MALEPRTSV
KJ-1v. It is very important that we get accurate responses to this key question. The computer recorded that you have had (MALEPRTS[1]) male sex partners in your entire life. If this answer is correct, select "Yes" to move on to the next question. If the computer recorded an incorrect response, please select "No" to change your answer.

Yes 1
No 5

FLOW CHECK K-14c: IF MALEPRTSV = NO, RETURN AND ASK MALEPRTS[2].
ELSE IF MALEPRTSV NE NO OR MALEPRTS[2]=RESPONSE, GO TO FLOW CHECK K-14d.

FLOW CHECK K-14d: DEFINE SAMLIFENUM: ("NUMBER OF SAME-SEX PARTNERS IN LIFETIME. (computed in FC K-14d)")

IF KJ-1v MALEPRTSV = 1 or DK or RF or (MALEPRTSV = 5 and KJ-1 MALEPRTS_2 = DK or RF), THEN SAMLIFENUM = KJ-1 MALEPRTS_1.
ELSE IF MALEPRTSV = 5 and MALEPRTS_2 NE DK or RF, THEN SAMLIFENUM = MALEPRTS_2.

CREATE AN ARRAY TO LOOP THROUGH MALPRT12[X] UP TO 2 TIMES.

MALPRT12[X]
KJ-2. Thinking about the last 12 months, how many male sex partners have you had in the 12 months since (CMLSTYR_FILL)? Please count every partner, even those you had sex with only once in those 12 months.

UNDERLYING RANGE: 0 to 995

FLOW CHECK K-14e: **IF MALPRT12[1] 11, 22, 33, 44, 55, 66, 77, 88,99, 111, 222,333, 444, 555, 666, 777, or 888) AND MALEPRTSV HAS NOT BEEN ASKED (R IS ONLY ASKED THIS ACASI ERROR ROUTING QUESTION ONCE),**
ASK MALPRT12V KJ-2v.
ONCE MALPRT12V NE NO AND MALPRT12[2]=RESPONSE, GO TO FLOW CHECK K-15.
ELSE GO TO KJ-3 SAMESEX1.

{ASKED ONCE IF FC K-14e IS TRUE AND MALEPRTSV HAS NOT BEEN ASKED
MALPRT12V
KJ-2v. It is very important that we get accurate responses to this key question. The computer recorded that you have had (MALPRT12[1]) male sex partners in the last 12 months. If this answer is correct, select "Yes" to move on to the next question. If the computer recorded an incorrect response, please select "No" to change your answer.

Yes 1
No 5

FLOW CHECK K-14f: **IF MALPRT12V = NO, RETURN AND ASK MALPRT12[2].**
ELSE IF MALPRT12V NE NO OR MALPRT12[2]=RESPONSE:

DEFINE SAMYEARNUM: (“NUMBER OF SAME-SEX PARTNERS IN LAST 12 MONTHS. (computed in FC K-14f)”)

IF KJ-2v MALPRT12V = 1 or DK or RF or (MALPRT12V = 5 and KJ-2 MALPRT12_2 = DK or RF), THEN SAMYEARNUM = KJ-2 MALPRT12_1.
ELSE IF MALPRT12V = 5 and MALPRT12_2 NE DK or RF, THEN SAMYEARNUM = MALPRT12_2.

THEN IF SAMYEARNUM=0 or sysmis, GO TO KJ-3 SAMESEX1.
ELSE IF 1 <= SAMYEARNUM <=995 THEN GO TO KJ-2YRa.

SAMORAL12
KJ-2YRa. ASK IF KH-1 GIVORALM=1 OR KH-2 GETORALM=1:
Your number of male partners in the last 12 months is displayed below. Thinking of your male partners in the last 12 months, with how many of them did you have oral sex?

DISPLAY: *[SAMYEARNUM] male partners in last 12 months*

UNDERLYING RANGE: 0 to 995

RECEPANAL12
KJ-2YRb. ASK IF KH-3 ANALSEX2=1:
(Your number of male partners in the last 12 months is displayed below.) Thinking of your male partners in the last 12 months, with how many of them did you have receptive anal sex where he put his penis in your anus (butt)?

DISPLAY: *[SAMYEARNUM] male partners in last 12 months*

UNDERLYING RANGE: 0 to 995

INSERANAL12

KJ-2YRc. ASK IF KH-4 ANALSEX3=1:
(Your number of male partners in the last 12 months is displayed below.) Thinking of your male partners in the last 12 months, with how many of them did you have insertive anal sex where you put your penis in his anus (butt)?

DISPLAY: *[SAMYEARNUM] male partners in last 12 months*

UNDERLYING RANGE: 0 to 995

SAMESEX1

KJ-3. Thinking back to the first time you ever had oral or anal sex with a male partner, how old were you?

UNDERLYING RANGE: 0 to 45

FLOW CHECK K-15: IF SAMYEARNUM > 0 or SAMYEARNUM = DK, ASK KJ-4 MSMNONMON.
ELSE IF SAMYEARNUM = 0 OR RF, GO TO KJ-11 CNDLSMAL.

MSMNONMON

KJ-4. Your number of male partners in the last 12 months is displayed below. In the last 12 months, that is, since (CMLSTYR_FILL), how many of your male partners were having sex with other people around the same time?

DISPLAY: *[SAMYEARNUM] male partners in last 12 months*

UNDERLYING RANGE: 0 to 995

MALSHT12

KJ-5. In the last 12 months, that is, since (CMLSTYR_FILL), have you had sex with a male who takes or shoots street drugs using a needle?

Yes 1
No 5

JOHN2FRQ

KJ-6. In the last 12 months, have you given a male money or drugs in exchange for having sex with you?

Yes 1
No 5

PROS2FRQ

KJ-7. In the last 12 months, has a male given you money or drugs to have sex with him?

Yes 1
No 5

HIVMAL12

KJ-8. In the last 12 months, have you had sex with a male who you knew was infected with the AIDS virus?

Yes 1
No 5

MSMWEB12

KJ-9. Some men meet their sexual partners by using the internet, and some do not. Internet includes the use of social network websites such as Facebook or MySpace, websites directed towards gay men such as Manhunt or Gay.com, dating websites, or the use of mobile social applications such as Foursquare or Grindr.

Thinking about your male sex partners in the last 12 months, did you first meet any of them using the internet?

Yes 1
No 5

MSMSORT12

KJ-10. Some men only have sex with other males that they know have the same HIV status as they do, and some do not. Thinking about your male sex partners in the last 12 months, do you usually limit your male partners to those of the same HIV status to prevent getting or transmitting HIV?

Would you say “yes, usually,” “yes, some of the time,” or “no”?

Yes, usually 1
Yes, some of the time ...3
No 5

{ Asked for all who have had sex with a male partner (samesexany=1)

CNDLSMAL

KJ-11. Now think of the last time you had oral or anal sex with a male partner, was a condom used?

Yes 1
No 5

Sexual Attraction, Orientation, & Experience with STDs (KK)

FLOW CHECK K-16: IF OPPSEXGEN=yes AND SAMESEXANY=yes, THEN DO:

IF R HAS REPORTED BOTH MALE AND FEMALE PARTNERS IN THE LAST 12 MONTHS ((OPPYEARNUM > 0 or OPPYEANUM = DK) and (SAMYEARNUM > 0 OR SAMYEARNUM = DK)), THEN ASK KK-1 CONDALLS.

ELSE IF R HAS REPORTED NO PARTNERS (male or female) IN THE LAST 12 MONTHS ((OPPYEARNUM = 0 or RF) and (SAMYEARNUM = 0 or RF)), THEN ASK KK-1 CONDALLS.

ELSE IF R HAS REPORTED ONLY FEMALE PARTNERS OR ONLY MALE PARTNERS IN THE LAST 12 MONTHS (OPPYEARNUM > 0 and (SAMYEARNUM = 0 or RF)) OR (SAMYEARNUM > 0 and (OPPYEARNUM = 0 or RF)), THEN GO TO KK-4 ATTRACT.

END DO.

ELSE IF R ONLY REPORTED SEXUAL ACTIVITY WITH FEMALES (involving male genitals) OR WITH MALES ((OPPSEXGEN=yes and SAMESEXANY=no) OR (OPPSEXGEN=no and SAMESEXANY=yes)), THEN

GO TO KK-4 ATTRACT.

CONDALLS
KK-1. The very last time you had any type of sex -- that is vaginal intercourse or anal sex or oral sex -- with a male or female partner, was a condom used?

Yes 1
No ....5 (KK-4 ATTRACT)

MFLASTP
KK-2. Was that last sexual partner male or female?

Male 1
Female 2

FLOW CHECK K-17: IF KK-2 MFLASTP = 2, ASK KK-3 WHYCOND.
ELSE IF MFLASTP = 1, DK, OR RF, GO TO KK-4 ATTRACT.

WHYCOND
KK-3. Was the condom used...

To prevent pregnancy 1
To prevent diseases like syphilis, gonorrhea or AIDS 2
For both reasons 3
Or for some other reason 4

ATTRACT
KK-4. People are different in their sexual attraction to other people. Which best describes your feelings? Are you...

Only attracted to females 1
Mostly attracted to females 2
Equally attracted to females and males 3
Mostly attracted to males 4
Only attracted to males 5
Not sure 6

ORIENT
KK-5. Do you think of yourself as ...

Heterosexual or straight 1
Homosexual or gay................2
Bisexual 3

INTROK16
KK-6. These next questions are about your sexual and reproductive health.

Please press [Enter] to continue.

TYPE: STRING [1]; ATTRIBUTES: EMPTY ALLOWED

{ Asked for all Rs
RISKCHEK1
KK-6a. In the last 12 months, that is, since (CMLSTYR_FILL), has a doctor or other medical care provider asked you about your sexual orientation or the sex of your sexual partners?

Yes 1
No 5

{ Asked for all Rs
RISKCHEK2
KK-6b. In the last 12 months, has a doctor or other medical care provider asked you about your number of sexual partners?

Yes 1
No 5

{ Asked for all Rs
RISKCHEK3
KK-6c. In the last 12 months, has a doctor or other medical care provider asked you about your use of condoms?

Yes 1
No 5

{ Asked for all Rs
RISKCHEK4
KK-6d. In the last 12 months, has a doctor or other medical care provider asked you about the types of sex you have, whether vaginal, oral, or anal?

Yes 1
No 5

FLOW CHECK K-17_0: **IF R IS 18 YEARS OR OLDER (AGE_R >= 18) AND R HAS HAD ANAL SEX WITH A MALE PARTNER IN THE PAST 12 MONTHS (0 < RECEPANAL12 < 995 OR 0 < INSERANAL12 < 995), ASK KK-6E RECTDOUCH.**

ELSE IF R IS YOUNGER THAN 18 (AGE_R < 18) OR R HAS NOT HAD ANAL SEX WITH A MALE PARTNER IN THE PAST YEAR (RECEPANAL12=SYSMIS, 0, DK/RF AND INSERANAL=SYSMIS, 0, DK/RF), GO TO KK-7 STDTST12.

{ Asked if R >=18 years and has had anal sex with male partner in last year
RECTDOUCH
KK-6e. Some men use a rectal douche before or after anal sex, and some do not. During the last 12 months, that is, since (CMLSTYR_FILL), how often, if at all, did you use a rectal douche?

Never 1
Once or twice during the year 2
Several times during the year 3
About once a month 4
About once a week 5
About once a day or more 6

{ Asked for all Rs
STDTST12
KK-7. In the past 12 months, that is, since (CMLSTYR_FILL), have you been tested by a doctor or other medical care provider for a sexually transmitted disease like gonorrhea, chlamydia, herpes, or syphilis?

Yes 1
No ....5 (GO TO KK-8 STDTRT12)

{ Asked only for Rs who said “yes” to STDTST12
STDSITE12
KK-7b. ASK IF KK-7 STDTST12 = 1 (YES):

In the past 12 months, have you been tested by a doctor or other medical care provider for a sexually transmitted disease like gonorrhea or chlamydia in your throat or pharynx or your rectum (anus or butt)?

Yes 1
No 5

STDTRT12

KK-8. In the past 12 months, have you been treated or received medication from a doctor or other medical care provider for a sexually transmitted disease like gonorrhea, chlamydia, herpes, or syphilis?

Yes 1
No 5

GON

KK-9. In the last 12 months, have you been told by a doctor or other provider that you had gonorrhea?

Yes 1
No 5

CHLAM

KK-10. In the last 12 months, have you been told by a doctor or other medical care provider that you had chlamydia?

Yes 1
No 5

HERPES

KK-11. At any time in your life, have you ever been told by a doctor or other medical care provider that you had genital herpes?

Yes 1
No 5

GENWARTS

KK-12. At any time in your life, have you ever been told by a doctor or other medical care provider that you had genital warts or human papillomavirus also called HPV?

Yes 1
No 5

SYPHILIS

KK-13. At any time in your life, have you ever been told by a doctor or other medical care provider that you had syphilis?

Yes 1
No 5

FLOW CHECK K-17_1: IF KC-6 INJECT12= 2, 3, or 4, GO TO KK-15 EVRSHARE. ELSE IF INJECT12= 1, DK, or RF, ASK KK-14 EVRINJECT.

EVRINJECT

KK-14. At any time in your life, have you ever shot up or injected drugs other than those prescribed for you

Yes 1
No ....5 (GO TO INTROK17)

EVRSHARE
KK-15. At any time in your life, have you ever shot up or injected drugs with a needle that someone else had used before you?

Yes 1
No 5

Individual Earnings and Family Income and Public Assistance (KL)

{ ASKED FOR ALL
INTROK17
KL-0. Income is important in analyzing the information we collect. For example, this information helps us to learn whether persons in each income group get the health services they need.

Please press [Enter] to continue.

TYPE: STRING [1]; ATTRIBUTES: EMPTY ALLOWED

FLOW CHECK K-17a: IF REARNTY = 5 THEN GO TO FLOW CHECK K-18.
ELSE, CONTINUE.

EARNTYPE
KL-0a. IF RWRKST = 1 (YES), ASK:
Next, please enter your total earnings before taxes. Will it be easier for you to enter your total earnings per week, per month, or per year?

IF RWRKST = 5 (NO), ASK:
Next, please enter your total earnings before taxes on your last job. Will it be easier for you to enter your total earnings per week, per month, or per year?

Week..............1
Month.............2
Year..............3

EARN
KL-0b. IF RWRKST = YES AND EARNTYPE = WEEKLY, ASK:
Which category represents your total weekly earnings before taxes?

IF RWRKST = NO AND EARNTYPE = WEEKLY, ASK:
Which category represents your total weekly earnings before taxes on your last job?

IF RWRKST = YES AND EARNTYPE = MONTHLY, ASK:
Which category represents your total monthly earnings before taxes?

IF RWRKST = NO AND EARNTYPE = MONTHLY, ASK:
Which category represents your total monthly earnings before taxes on your last job?

IF RWRKST = YES AND EARNTYPE = YEARLY, DK, OR RF, ASK:
Which category represents your total yearly earnings before taxes?

IF RWRKST = NO AND EARNTYPE = YEARLY, DK, OR RF, ASK:

Which category represents your total yearly earnings before taxes on your last job?

(WEEKLY INCOME CATEGORIES)
WEEKLY INCOME

UNDER $96	1
$ 96-143	2
$ 144-191	3
$ 192-239	4
$ 240-288	5
$ 289-384	6
$ 385-480	7
$ 481-576	8
$ 577-672	9
$ 673-768	10
$ 769-961	11
$ 962-1,153	12
$1,154-1,441	13
$1,442-1,922	14
$1,923 or more	15

(MONTHLY INCOME CATEGORIES)
MONTHLY INCOME

UNDER $417	1
$ 417-624	2
$ 625-832	3
$ 833-1,041	4
$1,042-1,249	5
$1,250-1,666	6
$1,667-2,082	7
$2,083-2,499	8
$2,500-2,916	9
$2,917-3,332	10
$3,333-4,166	11
$4,167-4,999	12
$5,000-6,249	13
$6,250-8,332	14
$8,333 or more	15

(YEARLY INCOME CATEGORIES)
YEARLY INCOME

UNDER $5,000	1
$ 5,000- 7,499	2
$ 7,500- 9,999	3
$10,000-12,499	4
$12,500-14,999	5
$15,000-19,999	6
$20,000-24,999	7
$25,000-29,999	8
$30,000-34,999	9
$35,000-39,999	10
$40,000-49,999	11
$50,000-59,999	12
$60,000-74,999	13
$75,000-99,999	14
$100,000 or more	15

FLOW CHECK K-17b: IF KL-0b EARN = DK OR RF, ASK KL-0c EARNDK1.
ELSE IF KL-0b EARN NE DK OR RF, GO TO FLOW CHECK K-18

EARNDK1
KL-0c. Was it $20,000 or more per year?

Yes 1

No ....5 (FLOW CHECK K-18)

EARNDK2
KL-0d. Was it $50,000 or more per year?

Yes 1
No ....5 (FLOW CHECK K-18)

EARNDK3
KL-0e. Was it $75,000 or more per year?

Yes 1
No ....5 (FLOW CHECK K-18)

EARNDK4
KL-0f. Was it $100,000 or more per year?

Yes 1
No 5

FLOW CHECK K-18: IF ROSCNT = 1, GO TO KL-1a WAGE.
ELSE IF ROSCNT > 1, CONTINUE.

INTROK18
KL-1. IF WOMREL = WIFE AND ROSCNT = 2, SAY:
The next questions are about your combined family income last year, that is, in the year [LASTYEAR_FILL]. When answering these questions, please remember that “combined family income” means your income <u>plus</u> your wife’s income, before taxes.

ELSE IF WOMREL = WIFE AND ROSCNT > 2, SAY:
The next questions are about your combined family income last year, that is, in the year [LASTYEAR_FILL]. When answering these questions, please remember that “combined family income” means your income <u>plus</u> your wife’s income, income from any of your family members that live here, and income from any of your wife’s family members that live here, before taxes.

ELSE IF WOMREL = FEMALE PARTNER AND ROSCNT = 2, SAY:
The next questions are about your combined family income last year, that is, in the year [LASTYEAR_FILL]. When answering these questions, please remember that “combined family income” means your income <u>plus</u> your partner’s income, before taxes.

ELSE IF WOMREL = FEMALE PARTNER AND ROSCNT > 2, SAY:
The next questions are about your combined family income last year, that is, in the year [LASTYEAR_FILL]. When answering these questions, please remember that “combined family income” means your income <u>plus</u> your partner’s income, and income from any of your family members that live here, before taxes.

ELSE IF WOMREL NE FEMALE PARTNER OR WIFE AND ROSCNT > 1, SAY:
The next questions ask about your combined family income last year, that is, in the year [LASTYEAR_FILL]. When answering these questions, please only include income from yourself and anyone else living here who is <u>related</u> to you. If there is no one living here who is related to you, “family” refers only to you.

Please press [Enter] to continue.

TYPE: STRING [1]; ATTRIBUTES: EMPTY ALLOWED

WAGE

KL-1a. IF ROSCNT > 1, ASK:
In the year [LASTYEAR_FILL], did you or any members of your family living here receive any wages and salaries, including tips, bonuses and overtime?

ELSE IF ROSCNT = 1, ASK:
In the year [LASTYEAR_FILL], did you receive any wages and salaries, including tips, bonuses and overtime?

Wages and salaries (including tips, bonuses, and overtime) are paid by employers in compensation for work performed by the employee. This includes wages to armed forces personnel.

Yes 1
No 5

SELFINC

KL-1b. IF ROSCNT > 1, ASK:
In the year [LASTYEAR_FILL], did you or any members of your family living here receive any income from self employment including business and farm income?

ELSE IF ROSCNT = 1, ASK:
In the year [LASTYEAR_FILL], did you receive any income from self employment including business and farm income?

{ To be displayed for all:
Self employment means being a full or part owner in a business or farm.

Yes 1
No 5

SOCSEC

KL-1c. IF ROSCNT > 1, ASK:
(In the year [LASTYEAR_FILL], did you or any members of your family living here receive...)

Any income from Social Security or Railroad Retirement?

ELSE IF ROSCNT = 1, ASK:
(In the year [LASTYEAR_FILL], did you receive ...)

Any income from Social Security or Railroad Retirement?

{ To be displayed for all:
Social Security retirement benefits are administered by the Social Security Administration and are paid to retired workers and their families.

Railroad Retirement benefits are administered by the Railroad Retirement Board and are paid to retired railroad workers and their families.

Yes 1
No 5

DISABIL

KL-1d. IF SOCSEC = YES AND ROSCNT > 1, ASK:

(In the year [LASTYEAR_FILL], did you or any members of your family living here receive...)

Any income from any disability pension other than Social Security or Railroad Retirement?

ELSE IF SOCSEC = YES AND IF ROSCNT = 1, ASK:
(In the year [LASTYEAR_FILL], did you receive...)

Any income from any disability pension other than Social Security or Railroad Retirement?

ELSE IF SOCSEC NE YES AND ROSCNT > 1, ASK:
(In the year [LASTYEAR_FILL], did you or any members of your family living here receive ...)

Any income from any disability pension?

ELSE IF SOCSEC NE YES AND IF ROSCNT = 1, ASK:
(In the year [LASTYEAR_FILL], did you receive...)

Any income from any disability pension?

Yes 1
No 5

RETIRE
KL-1e. IF SOCSEC = YES AND IF ROSCNT > 1, ASK:
(In the year [LASTYEAR_FILL], did you or any members of your family living here receive...)

Any income from any retirement or survivor pension other than Social Security or Railroad Retirement?

ELSE IF SOCSEC = YES AND IF ROSCNT = 1, ASK:
(In the year [LASTYEAR_FILL], did you receive...)

Any income from any retirement or survivor pension other than Social Security or Railroad Retirement?

ELSE IF SOCSEC NE YES AND ROSCNT > 1, ASK:
(In the year [LASTYEAR_FILL], did you or any members of your family living here receive...)
Any income from any retirement or survivor pension?

ELSE IF SOCSEC NE YES AND IF ROSCNT = 1, ASK:
(In the year [LASTYEAR_FILL], did you receive...)

Any income from any retirement or survivor pension?

Yes 1
No 5

SSI
KL-1f. IF ROSCNT > 1, ASK:
(In the year [LASTYEAR_FILL], did you or any members of your family living here receive...)

Any income from Supplemental Security Income (SSI)?

ELSE IF ROSCNT = 1, ASK:

(In the year [LASTYEAR_FILL], did you receive...)

Any income from Supplemental Security Income (SSI)?

{ To be displayed for all:

Supplemental Security Income is paid to persons aged 65 and over and to blind or disabled persons with incomes below specified levels. The benefits are administered by the Social Security Administration.

Yes 1
No 5

UNEMP

KL-1g. IF ROSCNT > 1, ASK:

(In the year [LASTYEAR_FILL], did you or any members of your family living here receive...)

Any income from unemployment compensation?

ELSE IF ROSCNT = 1, ASK:

(In the year [LASTYEAR_FILL], did you receive...)

Any income from unemployment compensation?

{ To be displayed for all:

Unemployment compensation is payment made by states to involuntarily unemployed workers who are able to work, available to work, and meet other state requirements.

Yes 1
No 5

CHLDSUPP

KL-1h. IF ROSCNT > 1, ASK:

(In the year [LASTYEAR_FILL], did you or any members of your family living here receive...)

Any income from child support?

ELSE IF ROSCNT = 1, ASK:

(In the year [LASTYEAR_FILL], did you receive...)

Any income from child support?
Yes 1
No 5

INTEREST

KL-1i. IF ROSCNT > 1, ASK:

(In the year [LASTYEAR_FILL], did you or any members of your family living here receive...)

Any income from interest from savings or other bank accounts?

ELSE IF ROSCNT = 1, ASK:

(In the year [LASTYEAR_FILL], did you receive...)

Any income from interest from savings or other bank accounts?

Yes 1
No 5

DIVIDEND
KL-1j. IF ROSCNT > 1, ASK:
(In the year [LASTYEAR_FILL], did you or any members of your family living here receive...)

Any income from dividends received from stocks or mutual funds, or net rental income from property, royalties, estates or trusts?

ELSE IF ROSCNT = 1, ASK:
(In the year [LASTYEAR_FILL], did you receive...)

Any income from dividends received from stocks or mutual funds, or net rental income from property, royalties, estates or trusts?

Yes 1
No 5

OTHINC
KL-1k. IF ROSCNT > 1, ASK:
In the year [LASTYEAR_FILL], did you or any members of your family living here receive any income from any other source, such as alimony, contributions from family or others, Veteran’s Administration (VA) payments, or Worker’s Compensation?

ELSE IF ROSCNT = 1, ASK:
In the year [LASTYEAR_FILL], did you receive any income from any other source, such as alimony, contributions from family or others, Veteran’s Administration (VA) payments, or Worker’s Compensation?

{ To be displayed for all:
Any other source could include alimony, VA payments, worker’s compensation, foster care payments, and other retirement income. Also include cash awards, education stipends, trust funds from other relatives, and anything else adding to family income.

Yes 1
No 5

TOINCWMY
KL-2. IF ROSCNT = 1, ASK:
The next question will ask about your <u>total</u> income in the year [LASTYEAR_FILL].

Remember, this item is important and your answers will be kept confidential. Will it be easier for you to report the total income per week, per month, or per year?

ELSE IF ROSCNT > 1, ASK:
The next question will ask about the <u>total combined income of your family</u> in the year [LASTYEAR_FILL].

Remember, this item is important and your answers will be kept confidential. Will it be easier for you to report the total income per week, per month, or per year?

Week..............1
Month.............2
Year..............3

TOTINC

KL-3. IF ROSCNT = 1 AND TOINCWMY = 1 (weekly), ASK:
Which category represents your total weekly income in the year [LASTYEAR_FILL], including income from all the sources you just went through, such as wages, salaries, Social Security or retirement benefits, help from relatives, and so forth? Please enter the amount before taxes.

ELSE IF ROSCNT = 1 AND TOINCWMY = 2 (monthly), ASK:
Which category represents your total monthly income in the year [LASTYEAR_FILL], including income from all the sources you just went through, such as wages, salaries, Social Security or retirement benefits, help from relatives, and so forth? Please enter the amount before taxes.

ELSE IF ROSCNT = 1 AND TOINCWMY = 3 (yearly), DK, OR RF, ASK:
Which category represents your total yearly income in the year [LASTYEAR_FILL], including income from all the sources you just went through, such as wages, salaries, Social Security or retirement benefits, help from relatives, and so forth? Please enter the amount before taxes.

ELSE IF ROSCNT > 1 AND TOINCWMY = 1, ASK:
Which category on represents the total combined weekly income of your family in the year [LASTYEAR_FILL], including income from all the sources you just went through, such as wages, salaries, Social Security or retirement benefits, help from relatives, and so forth? Please enter the amount before taxes.

ELSE IF ROSCNT > 1 AND TOINCWMY = 2, ASK:
Which category represents the total combined monthly income of your family in the year [LASTYEAR_FILL], including income from all the sources you just went through, such as wages, salaries, Social Security or retirement benefits, help from relatives, and so forth? Please enter the amount before taxes.

ELSE IF ROSCNT > 1 AND TOINCWMY = 3, DK, OR RF, ASK:
Which category represents the total combined yearly income of your family in the year [LASTYEAR_FILL], including income from all the sources you just went through, such as wages, salaries, Social Security or retirement benefits, help from relatives, and so forth? Please enter the amount before taxes.

IF ROSCNT > 1 and WOMREL = blank, DISPLAY:
Remember, only include income from yourself and anyone else living here who is related to you. If there is no one living here who is related to you, "family" refers only to you.

ELSE IF WOMREL = WIFE, DISPLAY:
Remember, "total combined income of your family" includes your income plus your wife's income, income from any of your family members that live here, and income from any of your wife's family members that live here, before taxes.

ELSE IF WOMREL = FEMALE PARTNER, DISPLAY:
Remember, "total combined income of your family" includes your income plus your partner's income, and income from any of your family members that live here, before taxes.

(WEEKLY INCOME CATEGORIES)
WEEKLY INCOME

UNDER $96 1
$ 96-143 2
$ 144-191 3
$ 192-239 4
$ 240-288 5
$ 289-384 6
$ 385-480 7
$ 481-576 8
$ 577-672 9
$ 673-768 10
$ 769-961 11
$ 962-1,153 12
$1,154-1,441 13
$1,442-1,922 14
$1,923 or more 15

(MONTHLY INCOME CATEGORIES)
MONTHLY INCOME

UNDER $417 1
$ 417-624 2
$ 625-832 3
$ 833-1,041 4
$1,042-1,249 5
$1,250-1,666 6
$1,667-2,082 7
$2,083-2,499 8
$2,500-2,916 9
$2,917-3,332 10
$3,333-4,166 11
$4,167-4,999 12
$5,000-6,249 13
$6,250-8,332 14
$8,333 or more 15

(YEARLY INCOME CATEGORIES)
YEARLY INCOME

UNDER $5,000 1
$ 5,000- 7,499 2
$ 7,500- 9,999 3
$10,000-12,499 4
$12,500-14,999 5
$15,000-19,999 6
$20,000-24,999 7
$25,000-29,999 8
$30,000-34,999 9
$35,000-39,999 10
$40,000-49,999 11
$50,000-59,999 12
$60,000-74,999 13
$75,000-99,999 14
$100,000 or more 15

FLOW CHECK K-19: IF KL-3 TOTINC = DK OR RF, ASK KL-3a FMINCDK1.
ELSE IF KL-3 TOTINC NE DK OR RF, GO TO FLOW CHECK K-20.

FLOW CHECK K-19a: CREATE POVTHRHLD_FILL (POVERTY THRESHOLD FILL BASED ON U.S. CENSUS BUREAU CALCULATIONS FOR THE POVERTY LEVEL INCOME BY FAMILY SIZE))

FMINCDK1
KL-3a. Was it less than $50,000 or $50,000 or more in (LASTYEAR_FILL)?

Less than $50,000..................1
$50,000 or more....................5 (FMINCDK4 KL-3d)

FLOW CHECK K-19aa: IF KL-3a FMINCDK1 = DK/RF, GO TO FLOW CHECK K-20.

{ ASKED IF INCOME WAS LESS THAN $50,000
FMINCDK2
KL-3b. Was it less than $35,000?

Yes 1
No 5

FLOW CHECK K-19ab: IF KL-3b FMINCDK2 = 5, DK/RF, GO TO FLOW CHECK K-20.

{ ASKED IF INCOME WAS LESS THAN $35,000
FMINCDK3
KL-3c. Was it less than (**POVTHRHLD_FILL**)?

Yes 1
No 5

FLOW CHECK K-19b: IF KL-3c = 1, 5, DK, OR RF, GO TO FLOW CHECK K-20.

{ ASKED IF INCOME WAS MORE THAN $50,000
FMINCDK4
KL-3d. Was it $75,000 or more last year?

Yes 1
No 5

FLOW CHECK K-19c: IF KL-3d FMINCDK4 = 5, DK/RF, GO TO FLOW CHECK K-20.

{ ASKED IF INCOME WAS MORE THAN $75,000
FMINCDK5
KL-3e. Was it $100,000 or more last year?

Yes 1
No 5

FLOW CHECK K-20: CREATE TANF_FILL (GENERATED FROM A LIST OF STATE TANF NAMES)

{ ASKED OF ALL
PUBASST
KL-4. At any time during [LASTYEAR_FILL], even for one month, did you or any members of your family living here receive any cash assistance from a state or county welfare program, such as (TANF_FILL)?

{ TO BE DISPLAYED FOR ALL:
Do not include Food Stamps, SSI, Energy Assistance, WIC, School Meals, or Transportation, Child Care, Rental or Education Assistance.

Yes 1
No ....5 (KL-6 FOODSTMP)

PUBASTYP
KL-5. From what type of program did you or any members of your family living here receive the cash assistance? Was it a welfare or welfare-to-work program such as (TANF_FILL), General Assistance, Emergency Assistance, or some other program?

Please enter all that apply.

To enter multiple answers, enter the number of the first answer, press the space bar, enter the number of the next answer, and so forth. The space bar is the long key at the bottom of the keyboard, in the middle. Press [Enter] once you're finished entering all your answers.

(TANF_FILL)/welfare/AFDC............................1
General Assistance..................................2
Emergency Assistance/short-term cash assistance......3
Some other program..................................4

FLOW CHECK K-21: CREATE SNAP_FILL FOR ALL (GENERATED FROM A LIST OF STATE SNAP PROGRAM CARD NAMES)

FOODSTMP
KL-6. The next question is about SNAP, the Supplemental Nutrition Assistance Program, formerly known as the Food Stamp Program. SNAP benefits are provided on an electronic debit card {called [SNAP_FILL]/or EBT card}. In the year [LASTYEAR_FILL], did you or any members of your family living here receive food stamps or SNAP benefits?

Yes 1
No 5

WIC
KL-7. In the year [LASTYEAR_FILL], did you or any members of your family living here receive WIC, the Women, Infants, and Children Nutrition Program?

Yes 1
No 5

HLPTRANS
KL-8a. In the year [LASTYEAR_FILL], did you or any members of your family living here receive the following type of government assistance because your income was low...

Transportation assistance, such as gas vouchers, bus passes, or help registering, repairing, or insuring a car?

Yes 1
No 5

HLPCHLDC
KL-8b. *(In the year [LASTYEAR_FILL], did you or any members of your family living here receive the following type of government assistance because your income was low ...)*

Any child care services or assistance so you or they could go to work or school or training?

Yes 1
No 5

HLPJOB
KL-8c. *(In the year [LASTYEAR_FILL], did you or any members of your family living here receive the following type of government assistance because your income was low ...)*

A social services or Welfare office's help with job training, a Job Club, a job search program, or anything else to help you or anyone in the household try to find a job?

Yes 1
No 5

{ ASKED FOR ALL
FREEFOOD
KL-9. In the last 12 months, did you receive free or reduced-cost food or meals because you couldn't afford to buy food?

Yes............1
No.............5

HUNGRY
KL-10. In the past 12 months, were you or any member of your family ever hungry, but you just couldn't afford more food?

Yes............1
No.............5

MED_COST
KL-11. In the past 12 months, was there anyone in your household who needed to see a doctor or go to the hospital but couldn't go because of the cost?

Yes............1
No.............5

Lock The responses you have given in this section will now be locked away to maintain your privacy. In order to activate the lock, please enter a number between 1 and 100 and press [Enter].

TYPE: INTEGER [3]

CONCLUSN
CONCLUSN. Thank you again for your participation in this study. Your responses to this special section have been successfully locked away. Please turn the computer back to the interviewer.

- *Interviewer: Please enter your code to continue.*

TYPE: INTEGER [4], ATTRIBUTES: NODK, NORF

Edit Check KL-12_1: IF CONCLUSN <> YEAR(SYSDATE), DISPLAY: You have entered the incorrect code, please re-enter.
HARD, NONSUPPRESSIBLE EDIT CHECK.

INTVCLOSE
INTVCLOSE. ☑ *Interviewer Checkpoint*

- *Please enter [1] to end the interview*

Complete............1

UNDERLYING RANGE: 1,NOEMPTY,NODONTKNOW,NOREFUSAL
